# Repetitive transcranial magnetic stimulation across neurodegenerative diseases: a systematic review and dose-response meta-analysis

**DOI:** 10.3389/fnagi.2025.1615734

**Published:** 2025-07-10

**Authors:** Yu Zhang, Yulin Wang, Ke Xu, Chengguang Zhang, Peizhu Lv, Yan Bai, Shun Wang

**Affiliations:** ^1^The Second Affiliated Hospital, Heilongjiang University of Chinese Medicine, Harbin Heilongjiang Province, China; ^2^Institute of Acupuncture and Moxibustion, Heilongjiang Academy of Traditional Chinese Medicine, Harbin, China

**Keywords:** repetitive transcranial magnetic stimulation, neurodegenerative diseases, dose-response meta-analysis, Parkinson’s disease, Alzheimer’s disease

## Abstract

**Objective:**

We summarized the existing clinical evidence of repetitive transcranial magnetic stimulation (rTMS) for Parkinson’s Disease (PD) and Alzheimer’s Disease (AD) and conducted a series of dose-response meta-analyses to determine the curve relationship between the number of pulses and the effect size of the treatment.

**Methods:**

Existing evidence was retrieved from five databases, and relevant outcome data on rTMS treatment for motor and non-motor symptoms of PD and AD were collected. Data were analyzed using R software to assess effect size using standardized mean differences (SMD) and 95% confidence intervals (CI). Heterogeneity testing was performed to assess differences in efficacy among the evidence, and restricted cubic spline (RCS) was used to fit the dose-response curves.

**Results:**

A total of 51 publications were included, involving 1,938 subjects. We found that for PD patients, the total number of rTMS pulses showed significant bell-shaped curves in TUG (χ^2^ = 6.87, df = 2, *p* = 0.03), FOGQ (χ^2^ = 15.17, df = 2, *p* = 0.001), BDI (χ^2^ = 14.33, df = 2, *p* = 0.001), HAMD (χ^2^ = 12.63, df = 2, *p* = 0.001), and HAMA (χ^2^ = 6.06, df = 2, *p* = 0.04). For AD patients, the total number of rTMS pulses demonstrated significant bell-shaped curves for MMSE (χ^2^ = 8.76, df = 2, *p* = 0.01) and MoCA (χ^2^ = 6.79, df = 2, *p* = 0.03).

**Conclusion:**

Our dose-response meta-analysis results show that rTMS demonstrates significant efficacy in certain motor and non-motor symptoms of PD and AD. The number of rTMS pulses presents a typical bell-shaped curve for these symptoms, indicating that more number of rTMS pulses is not always better; beyond a certain threshold, increasing the number of rTMS pulses correlates negatively with therapeutic efficacy.

## 1 Introduction

Neurodegenerative diseases (NDs) damage neurons in the nervous system over time. This damage results from multifactorial pathogenesis involving abnormal protein aggregation, neuroinflammation, abnormal neuronal death, and genetics (Hinz and Geschwind, 2017; Wilson et al., 2023). These processes affect the structure of synapses and neural networks, as well as normal connectivity and function, ultimately leading to impairments in motor, sensory, cognitive, emotional, linguistic, and social behaviors (Chi et al., 2018; Dugger and Dickson, 2017). As the global population ages, it is projected that approximately 10 million new cases of NDs will be reported each year. AD is currently the most common ND, accounting for 60–80% of all dementia cases (Duggan et al., 2020; Erkkinen et al., 2018). PD is the second most common ND after AD (Gonzales et al., 2022). Research indicates that in the next 30–40 years, the number of AD patients over 65 years old in the United States may exceed 10 million (Duggan et al., 2020; Golriz Khatami et al., 2020). The prevalence of PD in the population over 60 years old exceeds 10%, and about 40% of PD patients will experience non-motor symptoms or progress to dementia (Agnello and Ciaccio, 2022). Throughout the disease progression, both AD and PD often exhibit varying degrees of Motor or non-motor dysfunction, ultimately affecting patients’ families and social lives.

rTMS is a non-invasive electrophysiological tool that generates brief high-current pulses through a magnetic coil, which can alter the excitability of targeted brain areas and their interconnected regions, as well as changes in blood flow and neurotransmitter levels (Alzheimer’s and Dementia, 2023). Over the past 20 to 30 years, rTMS has developed into one of the important non-pharmacological treatment modalities for neurological and psychiatric disorders (Khan et al., 2019). Although the evidence-based guidelines for rTMS do not explicitly confirm its effectiveness for NDs, several recent studies have yielded relatively optimistic results. Recent randomized controlled trials and meta-analyses have shown that rTMS can significantly improve both motor and non-motor symptoms in subjects with AD and PD (Chou et al., 2022; Chung et al., 2020; Zhang et al., 2024). However, the effect size of rTMS is influenced by specific stimulation parameters, including duration, frequency, intensity, and stimulation target (Hoogendam et al., 2010; Pateraki et al., 2022). There is currently no consensus on the optimal rTMS parameters for clinical treatment of NDs (Wei et al., 2022). While some studies claim that a daily number of rTMS pulses less than 2000 falls within the safe range for rTMS, there is still no conclusion regarding a clear dose-response relationship between the number of rTMS pulses used for NDs and its efficacy (Anand and Hotson, 2002).

Increasingly, dose-response meta-analyses are being applied to the evidence of rTMS therapy (Sabé et al., 2024). These analyses can identify ineffective doses and the maximum or minimum number of rTMS pulses needed to achieve the maximum effect size by utilizing three different types of curves: ascending/descending curves, plateau curves, and bell-shaped curves. More importantly, they can help clinicians discover potential therapeutic effects of doses not yet explored in randomized controlled trials. Therefore, recognizing and understanding the dose-response relationship of rTMS parameters is crucial for guiding clinical practice. To address this gap, we conducted a series of dose-response meta-analyses on the clinical evidence of rTMS in PD and AD to examine the relationship between variations in specific parameters and the magnitude of treatment effects.

## 2 Methods

This systematic review and dose-response meta-analysis was conducted following the guidelines of the Preferred Reporting Items for Systematic Reviews and Meta-Analyses (PRISMA) (Liberati et al., 2009). The details of the PRISMA checklist can be found (see Supplementary material). The protocol for this systematic review has been registered with the International Registry of Prospective Systematic Reviews (PROSPERO) under registration number CRD42025635024.

### 2.1 Literature search and selection

The comprehensive search was conducted in the PubMed, Embase, Cochrane Library, OVID Medline, and Web of Science databases, with the search time set from establishing the databases until November 19, 2024. We combine subject keywords and free terms, structuring the search strategy into three parts: disease (Parkinson’s disease or Alzheimer’s disease), intervention (repetitive transcranial magnetic stimulation), and study type (randomized controlled trial). In addition, we carefully searched for meta-analyses related to the topic of this study and read the references in detail to ensure that all relevant information was included (see Supplementary material).

Two independent evaluators, ZY and XK, employed Endnote 20 to screen and review the literature, while author WS resolved any disputes arising from the literature screening process. The literature was initially screened based on titles and abstracts, after which eligible full texts of the studies were obtained for secondary screening.

### 2.2 Inclusion and exclusion criteria

We searched only evidence from studies published in peer-reviewed journals, and the PICOS principles were used to determine this study’s inclusion and exclusion criteria; manuscripts published only online were also included in our review. The inclusion criteria are as follows: (1) All RCTs (parallel or crossover design) must involve participants aged over 18 years who meet the diagnostic criteria for Parkinson’s disease or Alzheimer’s disease. (2) The study arms must include multiple number of rTMS pulses or compare the effects of a specific number of rTMS pulses with a placebo on outcomes. (3) The study must provide clear parameters regarding total number rTMS of pulses, stimulation sessions, daily number rTMS of pulses, and other stimulation parameters. (4) The study must evaluate improvements in motor or non-motor symptoms of Parkinson’s disease or Alzheimer’s disease.

The exclusion criteria encompassed duplicate studies, animal research, review articles, conference proceedings, non-English publications, and non-randomized controlled trials. Furthermore, this study specifically excluded Lewy body dementia (LBD) and Parkinson’s disease dementia (PDD). Additionally, intervention groups employing more than two combined therapeutic approaches were not included in the final analysis.

### 2.3 Outcomes

We consider the Unified Parkinson’s Disease Rating Scale III (UPDRS III) and Alzheimer’s Disease Assessment Scale-Cognition (ADAS-Cog) as the primary outcomes for Parkinson’s disease (PD) and Alzheimer’s disease (AD), respectively. Unified Parkinson’s Disease Rating Scale (UPDRS), Timed Up and Go Test (TUG), Freezing of Gait Questionnaire (FOGQ), Beck Depression Inventory (BDI), Montreal Cognitive Assessment (MoCA), Mini-Mental State Examination (MMSE), Hamilton Anxiety Rating Scale (HAMA), Hamilton Depression Rating Scale (HAMD), Clinical Dementia Rating (CDR), and Geriatric Depression Scale (GDS) are assessed as secondary outcomes to evaluate the motor or non-motor symptoms of PD or AD.

### 2.4 Data extraction

Two independent reviewers (ZY and XK) extracted data from eligible articles, including publication year, data source, gender, age, education level, treatment course, stimulation target, stimulation frequency, stimulation intensity, number of pulses, and the mean difference in the motor or non-motor symptoms performance of participants before and after the intervention along with its corresponding SD or SEM. If the study used different data forms, such as quartiles or confidence intervals, the data were converted according to the Cochrane Handbook (Cumpston et al., 2019). For studies that reported effect estimates graphically, a web plot digitizer^1^ was used to estimate the effect sizes from the graphs. When a study outcome was evaluated at multiple time points, we selected outcome data immediately after treatment. Data were cross-checked to minimize potential errors, and disagreements were resolved through discussion with the corresponding author (WS).

### 2.5 Quality assessment

Two reviewers, ZY and XK, utilized the Cochrane Risk of Bias Assessment Tool (Higgins et al., 2011) to assess the methodological quality of the included studies. The risk tool incorporates seven critical sources of bias, including selective bias, implementation bias, measurement bias, follow-up bias, reporting bias, and other biases. Each article was categorized as “low risk,” “high risk,” or “unclear risk” for each type of bias. We utilized the Grading of Recommendations, Assessment, Development, and Evaluation (GRADE) approach to evaluate the quality of evidence for both primary and secondary outcomes. Following the GRADE handbook(Guyatt et al., 2011), we conducted our quality assessment. The GRADEpro Guideline Development Tool (GDT) was employed to produce the results. In cases of disagreement, the corresponding author arbitrated the issues.

### 2.6 Statistical analysis

We performed all statistical calculations using R software (version 4.4.2). The standardized mean difference (SMD) and its 95% confidence interval (CI) were used as effect sizes for continuous variables. A dose-response meta-analysis was conducted using the doresmeta package developed by Crippa et al. (2019), fitting a one-stage restricted cubic spline (RCS) to evaluate the dose-response relationship between the total number of rTMS pulses and the improvement in motor and non-motor symptoms of PD and AD. Three fixed percentile knots (5, 50 and 90%) were set according to Orsini et al. (2012) recommendations. Using Crippa’s method (Crippa and Orsini, 2016), we calculated the impact of every additional 5000 total pulses on participants’ motor and non-motor symptoms. Heterogeneity was quantified using *I*^2^ and *p*-values. When the number of included studies was ≥ 10, Egger’s test and funnel plots were used to detect publication bias. A leave-one-out method was employed for sensitivity analysis to determine the robustness and reliability of the pooled results. *P* < 0.05 was assessed as statistically significant.

## 3 Result

### 3.1 Literature search

**FIGURE 1:**
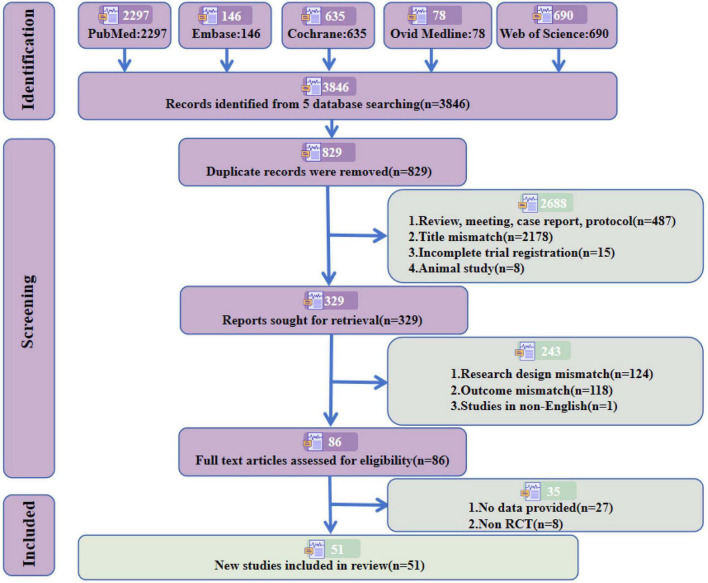
Flowchart of the screening process.

According to the previously established retrieval strategy, a total of 3,846 articles were retrieved (PubMed = 2,297, Embase = 146, Cochrane = 635, OVID Medline = 78, Web of Science = 690). After removing duplicate studies and strictly applying the inclusion and exclusion criteria, 51 studies meeting the required criteria were included, involving 1,938 subjects. Among these, 32 studies (Aftanas et al., 2018; Barboza et al., 2024; Benninger et al., 2011; Benninger et al., 2012; Boggio et al., 2005; Brys et al., 2016; Cardoso et al., 2008; Chang et al., 2016; Cohen et al., 2018; Grobe-Einsler et al., 2024; Huang et al., 2023; Ji et al., 2021; Jiang et al., 2023; Khedr et al., 2024; Khedr et al., 2003; Khedr et al., 2019; Kim et al., 2015; Lench et al., 2021; Li et al., 2020; Makkos et al., 2016; Maruo et al., 2013; Mi et al., 2019; Mitsui et al., 2022; Pal et al., 2010; Romero et al., 2024; Shimamoto et al., 2001; Shin et al., 2016; Song et al., 2024; Spagnolo et al., 2020; Wang et al., 2024; Wu et al., 2024; Zhuang et al., 2020) (63%) focused on PD, comprising 1,164 subjects, and 19 studies (Bagattini et al., 2020; Chen et al., 2023; Cotelli et al., 2011; Hoy et al., 2023; Jia et al., 2021; Jung et al., 2024; Koch et al., 2022; Lee et al., 2016; Leocani et al., 2020; Li et al., 2021; Padala et al., 2020; Saitoh et al., 2022; Vecchio et al., 2022; Wei et al., 2022; Wu et al., 2022; Wu et al., 2015; Yao et al., 2022; Zhao et al., 2017; Zhou et al., 2022) (37%) addressed AD, including 774 subjects. The detailed screening process and reasons for exclusion are presented in Figure 1 and Supplementary material.

### 3.2 Study characteristics

In the included studies, 49 adopted a parallel design (96%), while 2 employed a crossover design (4%). There were 842 female subjects (49%), and 6 studies did not report the gender ratio of subjects (203 subjects, 10%). 28 studies utilized a figure-of-8 coil (55%), 13 studies used other types of coils (25%), and 10 studies did not report the type of coil used (20%). The average total number of pulses was 23,300, with a range from 500 to 80,000. The average number of sessions was 14, with a range of 3 to 32. 28 studies (55%) involved single-target stimulation, while 23 studies (45%) involved multi-target stimulation. 5 studies reported stimulation frequencies of ≤ 1 Hz (10%), 28 studies had frequencies of 1–10 Hz (55%), and 18 studies reported frequencies > 10 Hz (35%). Detailed information on all included studies is reported in Supplementary Table 2.

### 3.3 Risk of bias assessment

**FIGURE 2:**
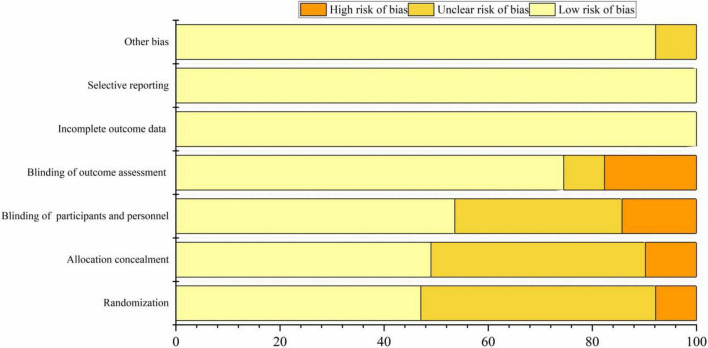
The risk of bias in the included studies.

The included 22 articles provided detailed descriptions of the randomization procedures. In 24 studies, only the randomization methods were reported, resulting in an assessment of “unknown risk.” Twenty studies employed appropriate methods to conceal allocation, such as using opaque envelopes. However, five studies did not clearly specify their allocation methods, which were considered “high risk.” Most studies conducted a sham rTMS procedure primarily using fake coils or adjusting coil positions, which ensured that the sounds heard by participants and the visual appearance of the coils were consistent with actual stimulation. As a result, 25 studies were assessed as having a “low risk” concerning the blinding of participants. 8 studies did not clearly describe the blinding procedures for assessors and were thus deemed to be at “high risk.” Specific sources of bias can be found in Figure 2 and Supplementary Table 1.

### 3.4 Effects of rTMS on PD

**FIGURE 3:**
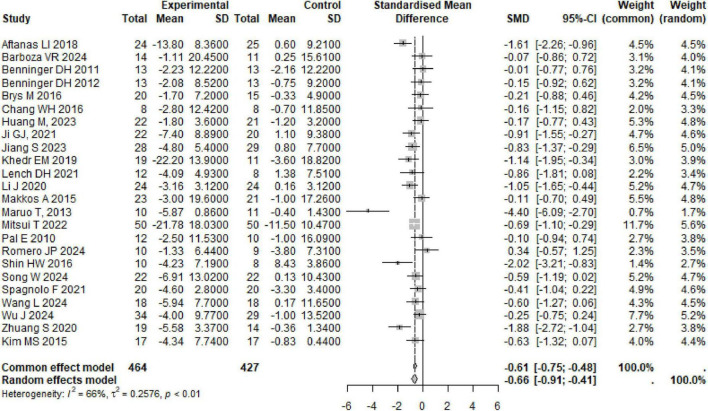
Forest plot of rTMS for UPDRS III.

A total of 24 studies were summarized on the improvement of UPDRS III scores in PD subjects through rTMS, involving 891 participants. The combined results showed that rTMS significantly reduced UPDRS III scores in PD subjects (SMD: −0.66, 95% CI [−0.91, −0.41], *p* < 0.01), with high heterogeneity among studies (*I*^2^ = 66%, *p* < 0.01) (Table 1 and Figure 3). We conducted a re-analysis based on differences in stimulation frequency, and the results showed: for stimulation frequency ≤ 1 Hz, the combined result was (SMD: −0.85, 95% CI [−1.53, −0.16], *p* = 0.01), with heterogeneity (*I*^2^ = 72%, *p* < 0.01); for 1–10 Hz (SMD: −0.68, 95% CI [−1.10, −0.26], *p* = 0.02), heterogeneity (*I*^2^ = 72%, *p* < 0.01); for > 10 Hz (SMD: −0.58, 95% CI [−0.98, −0.18], *p* = 0.04), with heterogeneity (*I*^2^ = 52%, *p* = 0.07); by stimulation target: Single Target Point (SMD: −0.80, 95% CI [−1.14, −0.47], *p* < 0.001), with heterogeneity (*I*^2^ = 58%, *p* < 0.01); Multiple Target Points (SMD: −0.55, 95% CI [−0.94, −0.15], *p* = 0.004), with heterogeneity (*I*^2^ = 72%, *p* < 0.01). Specific details can be found in Table 1 and Supplementary Figures 1–5. Using the leave-one-out method for sensitivity analysis, the results showed that the effect size and heterogeneity did not change significantly, indicating that the combined results are robust (see Supplementary Figures 22).

**TABLE 1:** Meta-analysis of the combined results of rTMS treatment for motor and non-motor symptoms of PD and AD and GRADE quality of evidence evaluation.

Items	No. of studies	No. of patients	Sex(F/M)	Mean of sessions (range)	Mean total pulse among included studies (range)	For the effect *P* value	SMD 95%CI	Heterogeneity (%)	Quality of the evidence (GRADE)
**rTMS for PD patients**									
UPDRS	24	891	362/479 NR:51	9(3, 14)	16,583(500, 80,000)	*P* = 0.005	−0.66(−0.91, −0.41)	66%	Moderate
≤ 1 Hz	4	152	69/83	10(10, 10)	13,000(12,000, 16,000)	*P* = 0.01	−0.85(−1.53, −0.16)	72%	–
1–10 Hz	14	524	234/269 NR:21	10(3, 20)	15,399(500, 80,000)	*P* = 0.02	−0.68(−1.10, −0.26)	72%	–
> 10 Hz	6	215	77/108 NR:30	8(5, 12)	21,733(2,400, 48,000)	*P* = 0.004	−0.58(−0.98, −0.18)	52%	−
Single target	11	398	174/194 NR:30	10(5, 14)	15,107(6,000, 40,000)	*P* < 0.001	−0.80(−1.14, −0.47)	58%	−
Multiple target	13	493	188/284 NR:21	8(3, 20)	16,669(500, 80,000)	*P* = 0.004	−0.55(−0.94, −0.15)	72%	−
UPDRS total score	13	552	180/327 NR:45	11(5, 24)	20,332(480, 80,000)	*P* = 0.02	−0.91(−1.98, 0.17)	86%	High
TUG	10	398	142/256	10(5, 24)	10,850(500, 25,200)	*P* < 0.0001	−0.41(−0.61, −0.21)	36%	Moderate
FOGQ	6	176	51/125	8(5, 10)	8,400(2400, 16,000)	*P* < 0.0001	−0.62(−0.92, −0.31)	0%	Moderate
HAMD	7	278	132/148	9(5, 10)	16,857(6,000, 48,000)	*P* = 0.02	−1.40(−2.71, −0.10)	90%	Moderate
BDI	7	214	65/119 NR:30	11(8, 24)	12,229(2,400, 40,000)	*P* < 0.0001	−0.61(−0.88, −0.33)	39%	Moderate
HAMA	4	192	94/98	8(5, 10)	20,000(10,000, 48,000)	*P* = 0.28	−0.78(−1.97, 0.40)	91%	Moderate
MoCA	3	140	65/75	10(10, 10)	10,000(6,000, 12,000)	*P* = 0.71	−0.12(−1.13, 0.89)	87%	Moderate
MMSE	5	155	62/72 NR:21	10(5, 12)	28,200(6,000, 48,000)	*P* = 0.10	0.26(−0.06, 0.58)	0%	Low
**rTMS for AD patients**									
ADAS-Cog	12	498	277/221	23(10, 32)	34,753(12,000, 60,000)	*P* = 0.03	−0.20(−0.38, −0.02)	28%	Moderate
≤ 10 Hz	7	261	154/107	21(10, 30)	29.691(12,000, 50,400)	*P* = 0.99	0.00(−0.25, 0.25)	0%	−
> 10 Hz	5	237	118/119	26(20, 32)	41,840(24,000, 60,000)	*P* = 0.002	−0.41(−0.67, −0.15)	9%	−
Single target	5	272	150/122	24(20,32)	39,440(24,000, 60,000)	*P* = 0.35	−0.22(−0.67, 0.23)	71%	−
Multiple target	7	226	117/109	22(10, 30)	31,406(12,000, 50,400)	*P* = 0.16	−0.19(−0.46,0.07)	0%	−
MMSE	14	541	292/239 NR:10	20(10, 32)	32,693(8,000, 60,000)	*P* = 0.04	0.43(0.02, 0.84)	80%	High
MoCA	6	185	102/83	19(10, 30)	27,867(12,000, 42,000)	*P* = 0.01	0.38(0.08, 0.67)	0%	High
CDR	4	205	132/73	18(10, 32)	24,800(8,000, 51,200)	*P* = 0.86	−0.03(−0.30, 0.25)	0%	Moderate
GDS	3	132	74/58	24(20, 30)	42,133(36,000, 50,400)	*P* = 0.73	0.06(−0.28, 0.41)	0%	Moderate

rTMS secondary outcomes in PD subjects covered various areas, including motor, emotion, and cognition, and showed significant improvements in multiple scores such as TUG, FOGQ, HAMD, and BDI. TUG (SMD: −0.41, 95% CI [−0.61, −0.21], *p* < 0.0001), heterogeneity (*I*^2^ = 36%, *p* = 0.12); FOGQ (SMD: −0.62, 95% CI [−0.92, −0.31], *p* < 0.0001), heterogeneity (*I*^2^ = 0%, *p* = 0.63); HAMD (SMD: −1.40, 95% CI [−2.71, −0.10], *p* = 0.04), heterogeneity (*I*^2^ = 90%, *p* < 0.01); BDI (SMD: −0.61, 95% CI [−0.88, −0.33], *p* < 0.0001), heterogeneity (*I*^2^ = 39%, *p* = 0.13). The improvement of UPDRS total score, HAMA, MoCA, and MMSE in subjects with PD following rTMS treatment does not seem to be significant. Specific details can be found in Table 1 and Supplementary Figures 6–13. The sensitivity analysis indicated that the effect sizes and heterogeneity for FOGQ, BDI, HAMD, and HAMA showed no significant changes, suggesting that the combined results are relatively robust. However, after excluding the studies by Aftanas LI, Mitsui T, and Makkos A, there were significant fluctuations in the effect sizes or heterogeneity of the combined results for UPDRS total score, TUG, and MoCA (see Supplementary Figures 23–30).

### 3.5 Effects of rTMS on AD

**FIGURE 4:**
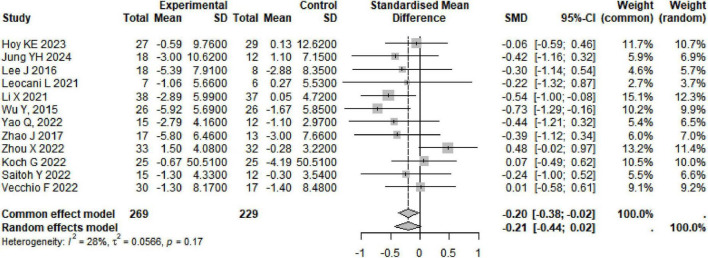
Forest plot of rTMS for ADAS-Cog.

A total of 12 studies were summarized on the improvement of ADAS-Cog scores in AD subjects through rTMS, involving 498 participants. The combined results showed that rTMS significantly reduced ADAS-Cog scores in AD subjects (SMD: −0.20, 95% CI [−0.38, −0.02], *p* = 0.03), with heterogeneity among studies (*I*^2^ = 28%, *p* = 0.17) (Table 1 and Figure 4). We conducted a re-analysis based on differences in stimulation frequency, and the results showed: for stimulation frequency ≤ 10 Hz, the combined result was (SMD: 0, 95% CI [−0.25, 0.25], *p* = 0.99), with heterogeneity (*I*^2^ = 0%, *p* = 0.43); for > 10 Hz (SMD: −0.41, 95% CI [−0.67, −0.15], *p* = 0.002), with heterogeneity (*I*^2^ = 9%, *p* = 0.36); by stimulation target: Single Target Point (SMD: −0.22, 95% CI [−0.67, −0.23], *p* = 0.35), with heterogeneity (*I*^2^ = 71%, *p* < 0.01); Multiple Target Points (SMD: −0.55, 95% CI [−0.46, −0.07], *p* = 0.16), with heterogeneity (*I*^2^ = 0%, *p* = 0.96) (Specific details can be found in Table 1 and Supplementary Figures 14–17). After excluding the study by Zhou X, there were fluctuations in the heterogeneity (see Supplementary Figures 31).

The combined results indicate that rTMS can significantly improve secondary outcome measures such as MMSE and MoCA in subjects with AD. MMSE (SMD: 0.43, 95% CI [0.02, 0.84], *p* = 0.03), heterogeneity (*I*^2^ = 80%, *p* < 0.01); MoCA (SMD: 0.38, 95% CI [0.08, 0.67], *p* = 0.01), heterogeneity (*I*^2^ = 0%, *p* = 0.96); The improvement of CDR and GDS in subjects with AD following rTMS treatment does not seem to be significant (Specific details can be found in Table 1 and Supplementary Figures 18–21). The sensitivity analysis indicated that the effect sizes and heterogeneity for MoCA, CDR, and GDS showed no significant changes, suggesting that the combined results are relatively robust. However, after excluding the studies by Li X, there was significant fluctuations in the heterogeneity of the combined results for MMSE (see Supplementary Figures 32–35).

### 3.6 Dose-response analysis

**FIGURE 5:**
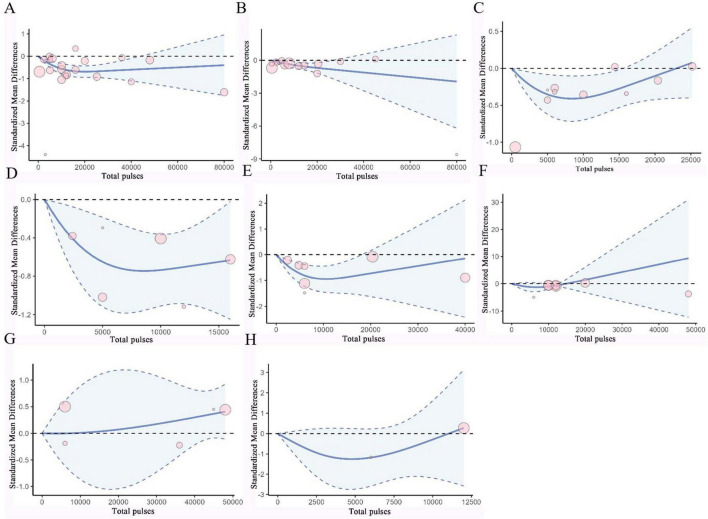
Dose-response curves of TMS for treating PD. **(A)** Dose-response relationship between total pulses and improvement of UPDRS III. **(B)** Dose-response relationship between total pulses and improvement of UPDRS. **(C)** Dose-response relationship between total pulses and improvement of TUG. **(D)** Dose-response relationship between total pulses and improvement of FOGQ. **(E)** Dose-response relationship between total pulses and improvement of BDI. **(F)** Dose-response relationship between total pulses and improvement of HAMD. **(G)** Dose-response relationship between total pulses and improvement of MMSE. **(H)** Dose-response relationship between Total pulses and improvement of MoCA. X-axis label, Number of rTMS pulses; Y-axis label, standardized mean difference; Blue curve, RCS curve; Blue dotted line, 95% CI; Pink circle, The included studies.

**FIGURE 6:**
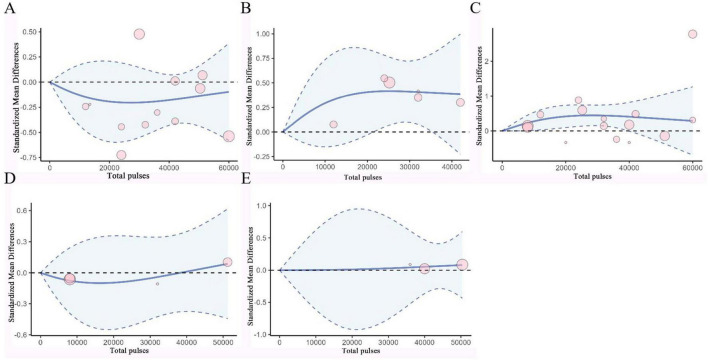
Dose-Response Curves of TMS for Treating AD. **(A)** Dose-response relationship between total pulses and improvement of ADAS-Cog. **(B)** Dose-response relationship between total pulses and improvement of MoCA. **(C)** Dose-response relationship between total pulses and improvement of MMSE. **(D)** Dose-response relationship between total pulses and improvement of CDR. **(E)** Dose-response relationship between total pulses and improvement of GDS. X-axis label, Number of rTMS pulses; Y-axis label, standardized mean difference; Blue curve, RCS curve; Blue dotted line, 95% CI; Pink circle, The included studies.

Based on RCS, a non-linear dose-response meta-analysis was conducted on the total number of pulses and the improvement of motor and non-motor symptoms in subjects with PD and AD. The results showed that the total number of rTMS pulses had significant bell-shaped curves for PD subjects in TUG (χ^2^ = 6.87, df = 2, *p* = 0.03), FOGQ (χ^2^ = 15.17, df = 2, *p* = 0.001), BDI (χ^2^ = 14.33, df = 2, *p* = 0.001), HAMD (χ^2^ = 12.63, df = 2, *p* = 0.001), and HAMA (χ^2^ = 6.06, df = 2, *p* = 0.04). Specifically, TUG achieved maximum therapeutic effect after receiving 8731 pulses (SMD: −0.41, 95% CI [−0.71, −0.10]); FOGQ achieved maximum effect after 8,763 pulses (SMD: −0.74, 95% CI [−1.14, −0.35]); BDI reached its peak effect after 11,535 pulses (SMD: −0.95, 95% CI [−1.47, −0.43]); HAMD after 7,705 pulses (SMD: −1.28, 95% CI [−2.83, 0.27]); and HAMA after 6,518 pulses (SMD: −0.78, 95% CI [−1.94, 0.37]). This indicates that an increase in total pulse rTMS correlates with improvements in motor or non-motor symptoms after receiving rTMS in the short term. A significant decreasing curve was observed for the UPDRS total score (χ^2^ = 12.14, df = 2, *p* = 0.002). Furthermore, for UPDRS III (χ^2^ = 27.58, df = 2, *p* = 1.02), MMSE (χ^2^ = 2.54, df = 2, *p* = 0.28), and MoCA (χ^2^ = 2.72, df = 2, *p* = 0.26), relatively flat bell-shaped curves were observed, indicating that the increase in total pulse numbers had no significant effect on the improvement of these outcomes. For AD subjects, the total number of rTMS pulses showed significant bell-shaped curves for MMSE (χ^2^ = 8.76, df = 2, *p* = 0.01) and MoCA (χ^2^ = 6.79, df = 2, *p* = 0.03), meaning that MMSE reached its maximum therapeutic effect after receiving 28793 pulses [SMD: 0.45, 95% CI (0.15, 0.74)] and MoCA after 25201 pulses [SMD: 0.42, 95% CI (0.07, 0.77)]. Additionally, no significant effects were observed for ADAS-Cog (χ^2^ = 1.93, df = 2, *p* = 0.38), CDR (χ^2^ = 0.28, df = 2, *p* = 0.87), and GDS (χ^2^ = 0.14, df = 2, *p* = 0.93), as they also displayed relatively flat bell-shaped curves(Specific details can be found in Figures 5, 6, and Supplementary Figures 36–44). In addition, we also presented the effect size and 95% confidence interval corresponding to the improvement in symptoms for every additional 5,000 total number of pulses (see Table 2).

**TABLE 2:** The effects of different pulse counts on motor and non-motor symptoms of PD and AD (standardized mean difference and 95% CI).

rTMS total pulse	0(ref)	5,000	10,000	15,000	20,000	25,000	30,000	35,000	40,000
UPDRS III	0	−0.35(−0.51, −0.19)	−0.56(−0.80, −0.32)	−0.66(−0.92, −0.41)	−0.69(−0.95, −0.43)	−0.68(−0.95, −0.40)	−0.65(−0.99, −0.32)	−0.63(−1.04, −0.21)	−0.60(−1.11, −0.10)
UPDRS total score	0	−0.24(−0.47, −0.02)	−0.43(−0.72, −0.14)	−0.58(−0.90, −0.25)	−0.70(−1.17, −0.22)	−0.80(−1.54, −0.07)	−0.91(−1.94, −0.13)	−1.01(−2.36, −0.34)	−1.11(−2.78,0.55)
TUG	0	−0.65(−0.62, −0.08)	−0.40(−0.70, −0.10)	−0.27(−0.53, −0.02)	−0.10(−0.42, 0.22)	0.07(−0.40, 0.54)	0.24(−0.41, 0.89)	0.41(−0.43, 1.25)	0.59(−0.45, 1.62)
FOGQ	0	−0.35(−1.12, −0.18)	−0.74(−1.11, −0.36)	−0.65(−1.19, −0.11)	−0.56(−1.51, 0.39)	−0.48(−1.88, 0.93)	−0.39(−2.26, 1.49)	−0.30(−2.64, 2.05)	−0.21(−3.03, 2.61)
BDI	0	−0.73(−1.12, −0.35)	−0.95(−1.45, −0.45)	−0.87(−1.50, −0.24)	−0.73(−1.62, 0.16)	−0.58(−1.79, 0.63)	−0.44(−1.99, 1.12)	−0.29(−2.20, 1.62)	−0.15(−2.42, 2.12)
HAMD	0	−1.20(−2.82, 0.41)	−1.03(−1.88, −0.18)	0.05(−1.67, 1.77)	1.45(−3.24, 6.13)	2.86(−4.84, 10.56)	4.27(−6.45, 14.99)	5.68(−8.06, 19.42)	7.10(−9.66, 23.86)
HAMA	0	−0.74(−1.90, 0.42)	−0.51(−0.92, −0.10)	0.62(−2.37, 3.60)	2.28(−4.94, 9.50)	4.15(−7.76, 16.06)	6.02(−10.60, 22.65)	7.90(−13.45, 29.25)	9.78(−16.29, 35.84)
MoCA	0	−1.25(−2.73, 0.24)	−0.29(−2.19, 1.61)	1.12(−3.37, 5.60)	2.53(−4.82, 9.87)	3.93(−6.31, 14.18)	5.34(−7.83, 18.51)	6.75(−9.34, 22.85)	8.16(−10.87, 27.19)
MMSE	0	−0.003(−0.53, 0.52)	0.009(−0.87, 0.89)	0.034(−1.03, 1.10)	0.072(−1.04, 1.19)	0.12(−0.93, 1.17)	0.17(−0.73, 1.08)	0.23(−0.47, 0.94)	0.30(−0.22, 0.82)
ADAS-Cog	0	−0.07(−0.25, 0.10)	−0.13(−0.43, −0.17)	−0.17(−0.54, 0.21)	−0.19(−0.59, 0.21)	−0.20(−0.59, 0.18)	−0.20(−0.55, 0.14)	−0.19(−0.49, 0.10)	−0.18(−0.43, 0.07)
MMSE	0	0.15(0.0007, 0.30)	0.28(0.016, 0.53)	0.36(0.050, 0.67)	0.42(0.095, 0.74)	0.44(0.14, 0.75)	0.44(0.15, 0.74)	0.43(0.10, 0.76)	0.41(−0.007, 0.82)
MoCA	0	0.17(−0.11, 0.45)	0.29(−0.15, 0.74)	0.37(−0.11, 0.85)	0.41(−0.03, 0.84)	0.42(0.07, 0.77)	0.41(0.10, 0.72)	0.40(0.01, 0.79)	0.39(−0.15, 0.93)
CDR	0	−0.05(−0.27, 0.16)	−0.09(−0.45, 0.27)	−0.10(−0.53, 0.34)	−0.09(−0.54, 0.36)	−0.08(−0.51, 0.35)	−0.05(−0.45, 0.34)	−0.02(−0.40, 0.35)	0.01(−0.37, 0.40)
GDS	0	0(−0.33, 0.33)	0.002(−0.62, 0.62)	0.006(−0.83, 0.84)	0.01(−0.92, 0.94)	0.02(−0.89, 0.93)	0.03(−0.76, 0.82)	0.04(−0.57, 0.65)	0.05(−0.36, 0.47)

### 3.7 Publication bias and grade quality of evidence

We conducted Egger’s test and generated a funnel plot for studies with more than 10 entries (see Supplementary Figures 45–48). The results showed: UPDRS III (*t* = −1.46, df = 22, *p* = 0.1581), UPDRS total score (*t* = −1.73, df = 11, *p* = 0.1111), TUG (*t* = 2.08, df = 8, *p* = 0.0713), ADAS-Cog (*t* = −0.66, df = 10, *p* = 0.5217), MMSE (*t* = 0.10, df = 12, *p* = 0.9222). These results suggest that there may not be significant publication bias.

We conducted an evaluation of the quality of evidence for the primary and secondary outcomes related to AD and PD. The results showed that there is high confidence in the evidence for the UPDRS total score, as well as the MMSE and MoCA scores of AD subjects. For UPDRS III, TUG, FOGQ, HAMD, BDI, HAMA, MoCA, ADAS-Cog, CDR, and GDS, the evidence levels maintain moderate confidence. However, there is limited confidence in the evidence level for the MMSE score results of PD subjects (Specific details can be found in Table 1 and Supplementary Table 3).

## 4 Discussion

The efficacy of rTMS is known to be frequency-dependent, as confirmed by numerous studies, but the impact of total number of pulses on diseases remains unknown (Sabé et al., 2024; Yu et al., 2024). To our knowledge, this is the first meta-analysis focusing on the dose-response relationship between number of rTMS pulses and motor and non-motor symptoms related to NDs. We collected all available evidence regarding rTMS for motor and non-motor symptoms in patients with PD and AD while applying the GRADE method to assess the certainty of the evidence in the included studies. We also discussed the significant dose-response relationship between number of rTMS pulses and symptom improvement.

In the rTMS parameter scheme for PD, we observed a significant bell-shaped curve between the total number of rTMS pulses and improvements in motor functions, such as the time to stand and walk and freezing of gait in PD patients. This indicates that a higher dose does not always result in better outcomes; beyond a certain threshold of pulse number, further stimulation may negatively correlate with symptom improvement. Some studies (Hsu et al., 2024) have pointed out that the best-fit curve for pulse number is bell-shaped, which aligns with our results. The combined results showed moderate evidence quality, and no significant heterogeneity was found. In non-motor symptoms of PD patients, such as depression and other emotional issues, a similar curve and trend were also observed. Likewise, a recent meta-analysis on the dose response of rTMS in treating psychiatric disorders observed a similar curve (Yu et al., 2024). We also noted a significant bell-shaped curve between pulse number and symptom improvement at stimulation frequencies of 1–10 Hz and with single-target stimulation, but no significant non-linear dose-response relationship was found in other stimulation frequencies and multi-target stimulation. This suggests that stimulation frequency and target act as important confounding factors in the relationship between pulse number and clinical efficacy. Furthermore, future clinical research could benefit from a greater focus on the relationship between rTMS stimulation parameter schemes and efficacy to develop reasonable rTMS protocols.

In the rTMS parameter scheme for AD, the total number of rTMS pulses showed a significant bell-shaped curve regarding cognitive function improvement in AD patients, with high evidence quality in the combined results. We found that only at frequencies greater than 10 Hz did the total number of rTMS pulses exhibit a significant bell-shaped dose-response relationship with efficacy. In contrast, we observed that the total number of rTMS pulses for PD patients’ UPDRS total score and AD patients’ MMSE, respectively, showed decreasing or increasing curves, indicating a positive correlation between providing more total pulses and symptom improvement. Therefore, future research could explore the feasibility and effectiveness of relevant rTMS protocols using more total pulses than those included in the current analysis.

Additionally, our combined results did not show significant positive results for rTMS on cognitive and anxiety symptoms in PD patients, nor for depressive symptoms in AD patients, and no significant dose-response relationship was observed. This contrasts with previously published research results (Xie et al., 2015; Zheng et al., 2022), which we believe may be partly explained by the limited number of qualifying studies. We advocate for more research to elucidate the dose-response relationship and therapeutic mechanisms of rTMS concerning these symptoms or to conduct more rigorous large-scale randomized controlled trials to determine its efficacy.

There are many different stimulation protocols for rTMS in clinical and research settings, which may contribute to heterogeneity in rTMS efficacy. Some studies have indicated that each stimulation parameter (number of rTMS pulses, intensity, target, frequency, etc.) is significantly related to efficacy (Hsu et al., 2024). Notably, in our study, the average total pulses for rTMS targeting AD patients exceeded 20,000 or even higher, with the need for 25,000 pulses or more to achieve maximum effect size. In another meta-analysis focusing on the dose-response of total pulse number in treating resistant depression, a high number of rTMS pulses was also reflected(Yu et al., 2024). In contrast, the average total pulse number for rTMS targeting PD patients’ symptoms was generally less than 20,000, with optimal efficacy achieved with as few as 10,000 pulses or even fewer. This is consistent with the evidence-based rTMS treatment guidelines updated in 2018, which mentioned the recommended pulse number for treating PD motor symptoms (Lefaucheur et al., 2020). However, whether the differences in the aforementioned number of rTMS pulses are due to differences in symptoms remains unknown.

## 5 Limitation

Our current study has certain limitations. First, we focused on two common neurodegenerative diseases, PD and AD, so it remains unclear whether our findings can be generalized to all degenerative diseases. Second, other stimulation parameters such as frequency, intensity, duration, and the initial severity of the disease, as well as factors like gender and age of the subjects, were not analyzed as confounding factors. Following the recommendation that each regression variable in meta-regression analysis should have at least 10 studies and considering the inconsistent reporting of potential regression variable data across all studies, we were unable to investigate the expected regression variables. Finally, the limited number of participants in some analyses resulted in non-significant dose-response curves for specific protocols.

## 6 Conclusion

Our dose-response meta-analysis results indicate that rTMS demonstrates significant efficacy in certain motor and non-motor symptoms of both PD and AD. The number of rTMS pulses exhibits a typical bell-shaped curve for some symptoms, suggesting that a higher number of pulses does not always yield better outcomes, which is consistent with previous studies. Additionally, this confirms the efficacy differences among rTMS protocols using varying pulse numbers. This finding encourages future clinical research to further examine the interactions between other stimulation parameters and to explore the dose-response relationships of rTMS in a broader range of degenerative diseases.

## Data Availability

The original contributions presented in the study are included in the article/Supplementary material, further inquiries can be directed to the corresponding authors.

## Footnotes

1
https://plotdigitizer.com
