# Supplementary material for: Repetitive transcranial magnetic stimulation across neurodegenerative diseases: a systematic review and dose-response meta-analysis
Source: Front Aging Neurosci. 2025 Jul 10;17:1615734. doi: 10.3389/fnagi.2025.1615734 (PMC12287036; doi:10.3389/fnagi.2025.1615734)
Supplement: Supplementary file 1 [file Data_Sheet_1.docx]

**Supplementary Material**

1. **PRISMA 2020 Checklist P2-P6**
2. **The complete search strategies P7-P9**
3. **Reasons for exclusion P10-P13**
4. **Risk of bias summary P14-P16**
5. **Characteristics of the included studies P17-P23**
6. **Grade Quality of evidence P24-P25**
7. **Other forest plot results P26-P31**
8. **Sensitivity Analysis P32-P36**
9. **Dose-response relationship P37-P40**
10. **Publication bias-funnel plot P41-P43**

| **eTable 1 PRISMA 2020 Checklist** | | | |
| --- | --- | --- | --- |
| **Section and Topic** | **Item #** | **Checklist item** | **Location where item is reported** |
| **TITLE** | | |  |
| Title | 1 | Identify the report as a systematic review. | P1 |
| **ABSTRACT** | | |  |
| Abstract | 2 | See the PRISMA 2020 for Abstracts checklist. | P1-2 |
| **INTRODUCTION** | | |  |
| Rationale | 3 | Describe the rationale for the review in the context of existing knowledge. | P2-3 |
| Objectives | 4 | Provide an explicit statement of the objective(s) or question(s) the review addresses. | P3 |
| **METHODS** | | |  |
| Eligibility criteria | 5 | Specify the inclusion and exclusion criteria for the review and how studies were grouped for the syntheses. | P4 |
| Information sources | 6 | Specify all databases, registers, websites, organisations, reference lists and other sources searched or consulted to identify studies. Specify the date when each source was last searched or consulted. | P4 |
| Search strategy | 7 | Present the full search strategies for all databases, registers and websites, including any filters and limits used. | P4 and Supplementary Material |
| Selection process | 8 | Specify the methods used to decide whether a study met the inclusion criteria of the review, including how many reviewers screened each record and each report retrieved, whether they worked independently, and if applicable, details of automation tools used in the process. | P4-5 |
| Data collection process | 9 | Specify the methods used to collect data from reports, including how many reviewers collected data from each report, whether they worked independently, any processes for obtaining or confirming data from study investigators, and if applicable, details of automation tools used in the process. | P5 |
| Data items | 10a | List and define all outcomes for which data were sought. Specify whether all results that were compatible with each outcome domain in each study were sought (e.g. for all measures, time points, analyses), and if not, the methods used to decide which results to collect. | P5 |
|  | 10b | List and define all other variables for which data were sought (e.g. participant and intervention characteristics, funding sources). Describe any assumptions made about any missing or unclear information. | P5 |
| Study risk of bias assessment | 11 | Specify the methods used to assess risk of bias in the included studies, including details of the tool(s) used, how many reviewers assessed each study and whether they worked independently, and if applicable, details of automation tools used in the process. | P5-6 |
| Effect measures | 12 | Specify for each outcome the effect measure(s) (e.g. risk ratio, mean difference) used in the synthesis or presentation of results. | P6 |
| Synthesis methods | 13a | Describe the processes used to decide which studies were eligible for each synthesis (e.g. tabulating the study intervention characteristics and comparing against the planned groups for each synthesis (item #5)). | P6 |
|  | 13b | Describe any methods required to prepare the data for presentation or synthesis, such as handling of missing summary statistics, or data conversions. | P5 |
|  | 13c | Describe any methods used to tabulate or visually display results of individual studies and syntheses. | P5-6 |
|  | 13d | Describe any methods used to synthesize results and provide a rationale for the choice(s). If meta-analysis was performed, describe the model(s), method(s) to identify the presence and extent of statistical heterogeneity, and software package(s) used. | P5 |
|  | 13e | Describe any methods used to explore possible causes of heterogeneity among study results (e.g. subgroup analysis, meta-regression). | P6 |
|  | 13f | Describe any sensitivity analyses conducted to assess robustness of the synthesized results. | P6 |
| Reporting bias assessment | 14 | Describe any methods used to assess risk of bias due to missing results in a synthesis (arising from reporting biases). | P5-6 |
| Certainty assessment | 15 | Describe any methods used to assess certainty (or confidence) in the body of evidence for an outcome. | P5-6 |
| **RESULTS** | | |  |
| Study selection | 16a | Describe the results of the search and selection process, from the number of records identified in the search to the number of studies included in the review, ideally using a flow diagram. | P6-7 |
|  | 16b | Cite studies that might appear to meet the inclusion criteria, but which were excluded, and explain why they were excluded. | P6-7 and Supplementary Material |
| Study characteristics | 17 | Cite each included study and present its characteristics. | Supplementary Material |
| Risk of bias in studies | 18 | Present assessments of risk of bias for each included study. | P7-8 |
| Results of individual studies | 19 | For all outcomes, present, for each study: (a) summary statistics for each group (where appropriate) and (b) an effect estimate and its precision (e.g. confidence/credible interval), ideally using structured tables or plots. | P8-10，Table1 and Supplementary Material |
| Results of syntheses | 20a | For each synthesis, briefly summarise the characteristics and risk of bias among contributing studies. | P8-10 |
|  | 20b | Present results of all statistical syntheses conducted. If meta-analysis was done, present for each the summary estimate and its precision (e.g. confidence/credible interval) and measures of statistical heterogeneity. If comparing groups, describe the direction of the effect. | P8-10 |
|  | 20c | Present results of all investigations of possible causes of heterogeneity among study results. | P8-10 |
|  | 20d | Present results of all sensitivity analyses conducted to assess the robustness of the synthesized results. | P8-10 |
| Reporting biases | 21 | Present assessments of risk of bias due to missing results (arising from reporting biases) for each synthesis assessed. | P8-10 |
| Certainty of evidence | 22 | Present assessments of certainty (or confidence) in the body of evidence for each outcome assessed. | P8-10 |
| **DISCUSSION** | | |  |
| Discussion | 23a | Provide a general interpretation of the results in the context of other evidence. | P11-12 |
|  | 23b | Discuss any limitations of the evidence included in the review. | P11-12 |
|  | 23c | Discuss any limitations of the review processes used. | P12-14 |
|  | 23d | Discuss implications of the results for practice, policy, and future research. | P12-14 |
| **OTHER INFORMATION** | | |  |
| Registration and protocol | 24a | Provide registration information for the review, including register name and registration number, or state that the review was not registered. | P4 |
|  | 24b | Indicate where the review protocol can be accessed, or state that a protocol was not prepared. | nr |
|  | 24c | Describe and explain any amendments to information provided at registration or in the protocol. | nr |
| Support | 25 | Describe sources of financial or non-financial support for the review, and the role of the funders or sponsors in the review. | P14 |
| Competing interests | 26 | Declare any competing interests of review authors. | P15 |
| Availability of data, code and other materials | 27 | Report which of the following are publicly available and where they can be found: template data collection forms; data extracted from included studies; data used for all analyses; analytic code; any other materials used in the review. | Supplementary Material |

**The complete search strategies**

**Cochrane**

Search Name:

Date Run: 18/11/2024 15:19:46

Comment:

ID Search Hits

#1 MeSH descriptor: [Parkinson Disease] explode all trees 6256

#2 (Parkinsonism):ti,ab,kw OR (Parkinson’s Disease):ti,ab,kw OR (PD):ti,ab,kw OR (Lewy Body Parkinson's Disease):ti,ab,kw OR (Idiopathic Parkinson's Disease):ti,ab,kw 51160

#3 #1 or #2 51160

#4 MeSH descriptor: [Alzheimer Disease] explode all trees 5489

#5 (Alzheimer Syndrome):ti,ab,kw OR (Alzheimer Diseases):ti,ab,kw OR (Senile Dementia):ti,ab,kw OR (Dementia):ti,ab,kw OR (AD):ti,ab,kw 41219

#6 #4 or #5 42433

#7 #3 or #6 90225

#8 (repetitive transcranial magnetic stimulation):ti,ab,kw OR (Transcranial Magnetic Stimulation):ti,ab,kw OR (Magnetic Stimulation):ti,ab,kw OR (rTMS):ti,ab,kw 12108

#9 #7 and #8 1184

#10 (randomized controlled trial):ti,ab,kw OR (randomized controlled):ti,ab,kw OR (clinic trial):ti,ab,kw OR (RCT):ti,ab,kw 915944

#11 #9 and #10 635

**Embase**

Session Results

.......................................................

No. Query Results Results Date

#11. #7 AND #9 AND #10 146 20 Nov 2024

#10. #3 OR #8 993,485 20 Nov 2024

#9. 'randomized controlled trial':ti,ab,kw OR 390,326 20 Nov 2024

'randomized controlled':ti,ab,kw OR 'clinic

trial':ti,ab,kw OR rct:ti,ab,kw

#8. #4 OR #5 566,496 20 Nov 2024

#7. 'transcranial magnetic stimulation':ti,ab,kw OR 33,613 20 Nov 2024

'magnetic stimulation':ti,ab,kw OR rtms:ti,ab,kw

#6. 'repetitive transcranial magnetic 6,800 20 Nov 2024

stimulation'/exp

#5. 'alzheimer syndrome':ti,ab,kw OR 'alzheimers 459,718 20 Nov 2024

diseases':ti,ab,kw OR 'senile dementia':ti,ab,kw

OR dementia:ti,ab,kw OR ad:ti,ab,kw

#4. 'alzheimer disease'/exp 269,234 20 Nov 2024

#3. #1 OR #2 480,643 20 Nov 2024

#2. parkinsonism:ti,ab,kw OR 'parkinsons 374,099 20 Nov 2024

disease':ti,ab,kw OR pd:ti,ab,kw OR 'lewy

body parkinsons disease':ti,ab,kw OR

'idiopathic parkinsons disease':ti,ab,kw

#1. 'parkinson disease'/exp 211,091 20 Nov 2024

.......................................................

**Ovid MEDLINE**

Ovid MEDLINE(R) and Epub Ahead of Print, In-Process, In-Data-Review & Other Non-Indexed Citations, Daily and Versions <1946 to November 19, 2024>

1 Parkinson Disease/ 88129

2 (Parkinsonism or PD or Parkinson's Disease).ab,ti,kw. 279531

3 Alzheimer Disease/ 129284

4 (Alzheimer Syndrome or Alzheimer's Diseases or Alzheimer Diseases or Senile Dementia or AD).ab,ti,kw. 199243

5 (repetitive transcranial magnetic stimulation or Transcranial Magnetic Stimulation or Magnetic Stimulation or rTMS).ab,ti,kw. 23476

6 (randomized controlled trial or randomized controlled or clinic trial or RCT).ab,ti,kw. 291882

7 1 or 2 293037

8 3 or 4 256332

9 7 or 8 538060

10 5 and 6 and 9 78

**Web of Science**

# Web of Science Search Strategy (v0.1)

# Database: All Databases

# Entitlements:

- WOS: 1900 to 2024

- BIOSIS: 1994 to 2024

- CSCD: 1989 to 2024

- DIIDW: 1966 to 2024

- FSTA: 1969 to 2024

- GRANTS: 1953 to 2024

- INSPEC: 1898 to 2024

- KJD: 1980 to 2024

- MEDLINE: 1950 to 2024

- PPRN: 1991 to 2024

- PQDT: 1637 to 2024

- SCIELO: 2002 to 2024

# Searches:

((TS=(Parkinson Disease OR Parkinson’s Disease OR Parkinsonism OR PD OR Lewy Body Parkinson's Disease OR Idiopathic Parkinson's Disease)) OR TS=(Alzheimer Syndrome OR Alzheimer's Diseases OR Alzheimer Diseases OR Senile Dementia OR Dementia OR AD)) AND TS=(repetitive transcranial magnetic stimulation OR Transcranial Magnetic Stimulation OR Magnetic Stimulation OR rTMS) AND TS=(randomized controlled trial OR randomized controlled OR clinic trial OR RCT) and Preprint Citation Index (Exclude – Database) Date Run: Wed Nov 20 2024 21:59:58 GMT+0800 (中国标准时间) Results: 690

**PubMed**

(((((((((((((Parkinson’s Disease) OR (Parkinson Disease)) OR (Parkinsonism)) OR (PD)) OR (Lewy Body Parkinson's Disease)) OR (Idiopathic Parkinson's Disease)) OR (Alzheimer's Diseases)) OR (Alzheimer Diseases)) OR (Alzheimer Syndrome)) OR (AD)) OR (Senile Dementia))) AND ((((repetitive transcranial magnetic stimulation) OR (Transcranial Magnetic Stimulation)) OR (Magnetic Stimulation)) OR (rTMS))) AND ((((randomized controlled trial) OR (randomized controlled)) OR (clinic trial)) OR (RCT)) 2297

**Reasons for exclusion**

**AD**

1.Anderkova L 2016

Distinct Pattern of Gray Matter Atrophy in Mild Alzheimer’s Disease Impacts on Cognitive Outcomes of Noninvasive Brain Stimulation

Exclude the cause：It is a comparative study of different stimulation sites.

2.Cotelli M 2008

Transcranial magnetic stimulation improves naming in Alzheimer disease patients at different stages of cognitive decline

Exclude the cause：No available outcome measures were included

3.Koch G, 2018

Transcranial magnetic stimulation of the precuneus enhances memory and neural activity in prodromal Alzheimer's disease
Exclude the cause：No available outcome measures were included

4.Leblhuber F 2022

High Frequency Repetitive Transcranial Magnetic Stimulation Improves Cognitive Performance Parameters in Patients with Alzheimer’s Disease – An Exploratory Pilot Study

Exclude the cause：Missing control data

5.Lin H 2024

Effects of accelerated intermittent theta-burst stimulation in modulating brain of Alzheimer’s disease

Exclude the cause：Missing data

6.Moussavi Z 2024

Repetitive transcranial magnetic stimulation as a treatment for Alzheimer's disease: A randomized placebo-controlled double-blind clinical trial

Exclude the cause：The resulting data are in the form of the difference from baseline

7. Saha C, 2024

Can Brain Volume-Driven Characteristic Features Predict the Response of Alzheimer’s Patients to Repetitive Transcranial Magnetic Stimulation? A Pilot Study

Exclude the cause：The research objectives do not match.

1. Tao Y 2022

Repetitive Transcranial Magnetic Stimulation Decreases Serum Amyloid-β and Increases Ectodomain of p75 Neurotrophin Receptor in Patients with Alzheimer’s Disease

Exclude the cause：Missing data

1. Teti Mayer J, 2021

Repetitive Transcranial Magnetic Stimulation as an Add-On Treatment for Cognitive Impairment in Alzheimer’s Disease and Its Impact on Self-Rated Quality of Life and Caregiver’s Burden

Exclude the cause：Missing control data

1. Zhang S 2023

Evaluating the treatment outcomes of repetitive transcranial magnetic stimulation in patients with

moderate-to-severe Alzheimer’s disease.

Exclude the cause：Missing data

1. Ahmed MA 2012

Effects of low versus high frequencies of repetitive transcranial magnetic stimulation on cognitive function and cortical excitability in Alzheimer’s dementia

Exclude the cause：Missing data

**PD**

1. Arias P 2010

Double-blind, randomized, placebo controlled trial on the effect of 10 days low-frequency rTMS over the vertex on sleep in Parkinson’s disease

Exclude the cause：Missing control data

1. Boylan LS 2001

Repetitive transcranial magnetic stimulation to SMA worsens complex movements in Parkinson's disease

Exclude the cause：Missing data

1. Dagan M 2017

The role of the prefrontal cortex in freezing of gait in Parkinson’s disease: insights from a deep repetitive transcranial magnetic stimulation exploratory study

Exclude the cause：non-rct

1. Eggers C 2015

Theta burst stimulation over the supplementary motor area in Parkinson’s disease

Exclude the cause：Grouping is not appropriate

1. Filipović SR, 2010

Low-frequency repetitive transcranial magnetic stimulation and off-phase motor symptoms in Parkinson's disease

Exclude the cause：Grouping is not appropriate

1. Hamada M 2008

High-Frequency rTMS over the Supplementary Motor Area for Treatment of Parkinson’s Disease

Exclude the cause：The resulting data are in the form of the difference from baseline

1. Hamada M 2009

High-frequency rTMS over the supplementary motor area improves bradykinesia in Parkinson's disease: Subanalysis of double-blind sham-controlled study

Exclude the cause：No outcome measures were available.

1. Hanajima R 2014

Triad-conditioning Transcranial Magnetic Stimulation in Parkinson’s Disease

Exclude the cause：No outcome measures were available.

1. Khedr EM 2007

Dopamine Levels After Repetitive Transcranial Magnetic Stimulation of Motor Cortex in Patients with Parkinson’s Disease: Preliminary Results

Exclude the cause：non-rct

1. Kim JY 2008

Therapeutic Effect of Repetitive Transcranial Magnetic Stimulation in Parkinson’s Disease: Analysis of [^11^C] Raclopride PET Study

Exclude the cause：non-rct

1. Mally J 1999

Improvement in Parkinsonian symptoms after repetitive transcranial magnetic stimulation

Exclude the cause：non-rct

1. Málly J 2004

Long-term follow-up study with repetitive transcranial magnetic stimulation (rTMS) in Parkinson’s disease

Exclude the cause：No outcome measures were available.

1. Okabe S 2003

0.2-Hz Repetitive Transcranial Magnetic Stimulation Has No Add-On Effects as Compared to a Realistic Sham Stimulation in Parkinson’s Disease

Exclude the cause：The resulting data are in the form of the difference from baseline

1. Okada KI 2021

Concomitant improvement in anti‑saccade success rate and postural instability gait

difculty after rTMS treatment for Parkinson’s disease

Exclude the cause：non-rct

1. Rektorova I 2007

Repetitive Transcranial Stimulation for Freezing of Gait in Parkinson’s Disease

Exclude the cause：non-rct

1. Chen J 2022

Comparative Analysis of the Effects of Escitalopram, Pramipexole, and Transcranial Magnetic Stimulation on Depression in Patients With Parkinson Disease: An Open-Label Randomized Controlled Trial

Exclude the cause：Pulse doses from RCTS were missing

1. Rektorova I 2008

Dorsolateral Prefrontal Cortex: A Possible Target for Modulating Dyskinesias in Parkinson’s Disease by Repetitive Transcranial Magnetic Stimulation

Exclude the cause：non-rct

1. Sayın S 2014

Low-frequency repetitive transcranial magnetic stimulation for dyskinesia and motor performance in Parkinson’s disease

Exclude the cause：No outcome measures were available.

1. Siebner HR 2000

Short-term motor improvement after sub-threshold 5-Hz repetitive transcranial magnetic stimulation of the primary motor hand area in Parkinson’s disease

Exclude the cause：non-rct

1. Tard C, 2016

Single session intermittent theta-burst stimulation on the left premotor cortex does not alleviate freezing of gait in Parkinson’s disease

Exclude the cause：No outcome measures were available.

1. Trung J, 2019

Transcranial magnetic stimulation improves cognition over time in Parkinson's disease

Exclude the cause：No outcome measures were available.

1. Liu S 2024

A Study on the Effects of Repetitive Transcranial Magnetic Stimulation on EEG Microstate in

Patients With Parkinson’s Disease

Exclude the cause：No outcome measures were available.

1. del Olmo MF 2007

Transcranial magnetic stimulation over dorsolateral prefrontal cortex in Parkinson’s disease

Exclude the cause：No outcome measures were available.

1. Lang S 2020

Theta-Burst Stimulation for Cognitive Enhancement in Parkinson’s Disease With Mild Cognitive Impairment: A Randomized, Double-Blind, Sham-Controlled Trial

Exclude the cause：No outcome measures were available.

| **eTable 2. Risk of bias summary.** | | | | | | | |
| --- | --- | --- | --- | --- | --- | --- | --- |
|  | **Random sequence generation (selection bias)** | **Allocation concealment (selection bias)** | **Blinding of participants and personnel (performance bias)** | **Blinding of outcome assessment (detection bias)** | **Incomplete outcome data (attrition bias)** | **Selective reporting (reporting bias)** | **Other bias** |
| Aftanas LI 2018 |  |  |  |  |  |  |  |
| Bagattini C 2020 |  |  |  |  |  |  |  |
| Barboza VR 2024 |  |  |  |  |  |  |  |
| Benninger DH 2011 |  |  |  |  |  |  |  |
| Benninger DH 2012 |  |  |  |  |  |  |  |
| Boggio PS 2005 |  |  |  |  |  |  |  |
| Brys M 2016 |  |  |  |  |  |  |  |
| Cardoso EF 2008 |  |  |  |  |  |  |  |
| Chang WH 2016 |  |  |  |  |  |  |  |
| Chen HF 2023 |  |  |  |  |  |  |  |
| Cohen OS 2018 |  |  |  |  |  |  |  |
| Cotelli M 2010 |  |  |  |  |  |  |  |
| Grobe-Einsler M 2024 |  |  |  |  |  |  |  |
| Hamada M 2008 |  |  |  |  |  |  |  |
| Hoy KE 2023 |  |  |  |  |  |  |  |
| Huang M, 2023 |  |  |  |  |  |  |  |
| Ji GJ, 2021 |  |  |  |  |  |  |  |
| Jia Y 2021 |  |  |  |  |  |  |  |
| Jiang S 2023 |  |  |  |  |  |  |  |
| Jung YH 2024 |  |  |  |  |  |  |  |
| Khedr EM 2003 |  |  |  |  |  |  |  |
| Khedr EM 2019 |  |  |  |  |  |  |  |
| Khedr EM 2024 |  |  |  |  |  |  |  |
| Kim MS 2015 |  |  |  |  |  |  |  |
| Koch G 2022 |  |  |  |  |  |  |  |
| Lee J 2016 |  |  |  |  |  |  |  |
| Lench DH 2021 |  |  |  |  |  |  |  |
| Leocani L 2021 |  |  |  |  |  |  |  |
| Li J 2020 |  |  |  |  |  |  |  |
| Li X 2021 |  |  |  |  |  |  |  |
| Makkos A 2015 |  |  |  |  |  |  |  |
| Maruo T 2013 |  |  |  |  |  |  |  |
| Mitsui T 2022 |  |  |  |  |  |  |  |
| Padala PR 2020 |  |  |  |  |  |  |  |
| Pal E 2010 |  |  |  |  |  |  |  |
| Romero JP 2024 |  |  |  |  |  |  |  |
| Saitoh Y 2022 |  |  |  |  |  |  |  |
| Shimamoto H 2001 |  |  |  |  |  |  |  |
| Shin HW 2016 |  |  |  |  |  |  |  |
| Song W 2024 |  |  |  |  |  |  |  |
| Spagnolo F 2021 |  |  |  |  |  |  |  |
| Vecchio F 2022 |  |  |  |  |  |  |  |
| Wang L 2024 |  |  |  |  |  |  |  |
| Wei L 2022 |  |  |  |  |  |  |  |
| Wu J 2024 |  |  |  |  |  |  |  |
| Wu X 2022 |  |  |  |  |  |  |  |
| Wu Y 2015 |  |  |  |  |  |  |  |
| Yao Q 2022 |  |  |  |  |  |  |  |
| Zhao J 2017 |  |  |  |  |  |  |  |
| Zhou X 2022 |  |  |  |  |  |  |  |
| Zhuang S 2020 |  |  |  |  |  |  |  |

Green: low risk, Yellow: unclear risk, Red: high risk.

| **eTable3. Characteristics of the included studies.** | | | | | | | | | | | |
| --- | --- | --- | --- | --- | --- | --- | --- | --- | --- | --- | --- |
| **References** | **Country** | **Sample(/F)** | **Age** | **HY Sacle/Education years(y)** | **Coil type** | **Target** | **Hz/(%MT)** | **Arm** | **Number of sessions (weeks)** | **Measured outcome** | **Dose** |
| Aftanas LI 2018 | Russia | C: 25/15 E: 24/11 | C: 63.8 ± 7.5 E: 63.2 ± 8.33 | Ⅱ-Ⅲ | NR | M1 and left DLPFC | 10Hz(100-110%) | C:Sham rTMS E:rTMS | 20 | UPDRS,UPDRS-III | 80000 |
| Barboza VR 2024 | Brazil | C: 11/7 E:14/7 | C: 58.4 ± 9.0 E: 52.6 ± 9.3 | NR | double-cone coil | posterior-superior insula(PSI) | 10Hz(80%) | C:Sham rTMS E:rTMS | 12(8 weeks) | UPDRSⅢ，MMSE,HAMA,HAMD | 36000 |
| Benninger DH 2011 | Switzerland | C:13/2 E:13/6 | C: 65.6 ± 9.0 E: 62.1 ± 6.9 | Ⅱ-Ⅳ | circular coil | M1 and DLPFC | 50Hz(80%) | C:Sham iTBS E: iTBS | 8(2 weeks) | UPDRS,UPDRS-III,BDI | 4800 |
| Benninger DH 2012 | Switzerland | C:13/4 E:13/2 | C: 63.7 ± 8.3 E: 64.5± 9.1 | Ⅱ-Ⅳ | circular coil | bilateral M1 | 50Hz(80%) | C:Sham rTMS E:rTMS | 8(2 weeks) | UPDRS,UPDRSIII,,BDI,FOGQ | 2400 |
| Boggio PS 2005 | Brazil | C:12 E:12 | T: 65.2 ± 8.10 | NR | figure-of-8 coil | left DLPFC | 15Hz(110%) | C:Sham rTMS and fluoxetine E: rTMS and placebo | 10(2 weeks ) | UPDRS | 30000 |
| Brys M 2016 | USA | C: 15/4 E: 20/9 | C: 64.0 ± 7.4 E: 64.9 ± 8.0 | II–IV | figure-of-8 coil | M1 and left DLPFC | 10Hz | C:Sham rTMS E:rTMS | 10(10 days) | UPDRS III,HAMD | 20000 |
| Cardoso EF 2008 | Brazil | C: 10 E: 11 | C: 63 ± 7.1 E: 67±8.3 | I-Ⅳ | figure-of-8 coil | left DLPFC | 5Hz(120%) | C:Sham rTMS and fluoxetine E: rTMS and placebo | 12(4 weeks) | UPDRS,MMSE | 45000 |
| Chang WH 2016 | Korea | T: 8/2 | C: T:71.9 ±7.8 | NR | double-cone coil | M1-LL | 10Hz(90%) | C:Sham rTMS E:rTMS | 5 (1 week) | UPDRS-III,TUG,FOGQ | 5000 |
| Cohen OS 2018 | Israel | C: 21/6 E: 21/4 | C: 66.8 ± 8.1 E: 64.4 ± 6.8 | Ⅱ-Ⅳ | H-coil | M1 and PFC | 1Hz(110%),10Hz(100%) | C:Sham rTMS E:rTMS | 24(12 weeks) | UPDRS,BDI,TUG | 20400 |
| Grobe-Einsler M 2024 | Germany | C: 17/4 E: 18/3 | C: 70.41 ± 10.37 E: 66.06 ± 9.70 | Ⅰ-Ⅲ | figure-of-8 coil | left lateral cerebellum and median cerebellum right lateral cerebellum | 48Hz(50%) | C:Sham rTMS E:rTMS | 10(5 day) | UPDRS,TUG | 14400 |
| Huang M, 2023 | China | C: 21/9 E: 22/8 | C: 69.6±8.2 E: 66.7±9.6 | Ⅰ-Ⅴ | figure-of-8 coil | midpoint of the sacral bone | 20Hz(40-60%) | C:Sham rTMS E:rTMS | 5(4 weeks) | UPDRS-III, MMSE, HAMA,HAMD | 48000 |
| Ji GJ, 2021 | China | C: 20/6 E: 22/8 | C: 60.2 ± 8.81 E: 61.7 ± 7.36 | Ⅰ-Ⅲ | figure-of-8 coil | left SMA | 50Hz(80%) | C:Sham cTBS E: iTBS | 14(2 weeks) | UPDRS-III,TUG | 25200 |
| Jiang S 2023 | China | C: 29/17 E: 28/14 | C: 64.3 ± 8.9 E: 62.7 ± 12.9 | I-Ⅳ | figure-of-8 coil | left DLPFC | 10Hz(100%) | C:Sham rTMS E:rTMS | 10(10 days) | UPDRS-III,HAMD,HAMA | 12000 |
| Khedr EM 2003 | Egypt | C: 19/5 E: 17/7 | C: 57.5 ± 8.4 E: 57.8 ± 9.2 | Ⅱ-Ⅲ | figure-of-8 coil | right then left hemispheres and EDB | 5Hz(120%) | C:Sham rTMS E:rTMS | 10(10 days) | UPDRS | 20000 |
| Khedr EM 2019 | Egypt | C:11 E:19 | C: 57.4 ± 10.0 E: 60.7 ± 8.8 | Ⅱ-Ⅳ | figure-of-8 coil | hand area of each motor cortex | 20Hz(90%) | C:Sham rTMS E:rTMS | 10(2 weeks) | UPDRS III | 40000 |
| Khedr EM 2024 | Egypt | C: 16/9 E: 8/2 | C: 60.21 ± 1.64 E: 61.82 ± 3.48 | NR | NR | right then left parietal areas | 20Hz(80%) | C:Sham rTMS E:rTMS | 10(2 weeks) | BDI | 40000 |
| Kim MS 2015 | Korea | T: 17/5 | T: 64.5 ± 8.4 | Ⅱ-Ⅲ | double-cone coil | M1-LL | 10Hz(90%) | C:Sham rTMS E:rTMS | 5 | UPDRS-III,TUG,FOGQ | 5000 |
| Lench DH 2021 | USA | C:8/1 E:12/5 | C: 64.5±8.9 E: 66.6±7.5 | NR | figure-of-8 coil | SMA | 1Hz(120%) | C:Sham rTMS E:rTMS | 10(10 days) | UPDRS-III,FOGQ | 12000 |
| Li J 2020 | China | C: 24/16 E: 24/16 | C: 61.46 ± 8.40 E: 61.67 ± 6.92 | I-Ⅳ | figure-of-8 coil | M1 | 20Hz(80%) | C:Sham rTMS E:rTMS | 5 | UPDRS-III,HAMD,HAMA | 10000 |
| Makkos A 2015 | Hungary | C: 21/10 E: 23/10 | C: 66 ± 5.93 E: 67 ± 9.63 | Ⅰ-Ⅳ | circular coil | bilateral M1 | 5Hz(90%) | C:Sham rTMS E:rTMS | 10(10 days) | UPDRS,UPDRS-III,,BDI,MMSE,MoCA,TUG | 6000 |
| Maruo T, 2013 | Japan | C: 11 E:10 | T: 63.0 ± 11.3 | Ⅱ-Ⅳ | NR | bilateral M1 foot area | 10Hz(100%) | C:Sham rTMS E:rTMS | 3 | UPDRS-III | 3000 |
| Mitsui T 2022 | Japan | C: 50/27 E: 50/26 | C: 70.98 ±7.30 E: 68.66 ± 8.69 | Ⅲ-Ⅳ | butterfy coil | spine Th12-L1 | 5Hz | C:Sham rTMS E:rTMS | 8（4 weeks） | UPDRS,TUG,UPDRS III | 500 |
| Pal E 2010 | Hungary | C: 10/5 E: 12/6 | C: 68.5 ± 7.78 E: 67.5 ± 11.11 | Ⅰ-Ⅱ | figure-of-8 coil | left DLPFC | 5Hz(90%) | C:Sham rTMS E:rTMS | 10(10 days) | UPDRS-III, BDI,TUG, MMSE | 6000 |
| Romero JP 2024 | Spain | C: 9/3 E: 10/3 | C: 66.89±9.07 E: 64.40±6.38 | Ⅰ-Ⅲ | figure-of-8 coil | bilateral M1 | 10Hz(80%) | C: not receive any therapy E:rTMS | 8(2 weeks) | UPDRS-III,TUG | 16000 |
| Shimamoto H 2001 | Japan | C: 9/4 E: 9/2 | C: 64.5±6.6 E: 65.1 ± 8.0 | I-Ⅳ | circular coil | frontal area | 0.2Hz | C:Sham rTMS E:rTMS | 8(8 weeks) | UPDRS | 480 |
| Shin HW 2016 | Korea | C: 8/6 E: 10/4 | C: 67 (54–79) E: 69 (55–82) | Ⅱ-Ⅲ | butterfy coil | left DLPFC | 5Hz(90%) | C:Sham rTMS E:rTMS | 10(2 weeks ) | UPDRS-III,HAMD,BDI | 6000 |
| Song W 2024 | China | C: 22/7 E: 22/7 | C: 70.50±6.76 E: 67.36±6.99 | Ⅱ-Ⅳ | figure-of-8 coil | bilateral M1 | 10Hz(90%) | C:Sham rTMS E:rTMS | 10(10 days) | FOGQ,TUG,UPDRS III,HAMD, HAMA | 10000 |
| Spagnolo F 2021 | Italy | C: 20/6 E: 20/5 | C: 62.4 ±5.5 E: 60.4 ±8.1 | Ⅱ-Ⅳ | H-coil | M1 | 10Hz(90%) | C:Sham rTMS E:rTMS | 12(weeks) | UPDRS-III | 10080 |
| Wang L 2024 | China | C: 18/10 E: 18/8 | C: 73.39 ± 6.15 E: 70.33 ± 9.36 | Ⅱ-Ⅲ | double-cone coil | bilateral M1-LL | 1Hz(120%) | C:Sham rTMS E:rTMS | 10(2 weeks ) | UPDRS-III,FOGQ | 16000 |
| Wu J, 2024 | China | C: 29/15 E: 34/15 | C: 65 ± 7.04 E: 63.0 ± 11.33 | Ⅱ-Ⅲ | figure-of-8 coil | right DLPFC | 1Hz(80%) | C:Sham rTMS E:rTMS | 10(10 days) | UPDRS III,MoCA | 12000 |
| Zhuang S, 2020 | China | C: 14/7 E: 19/8 | C: 61.57 ± 13.25 E: 60.58 ±9.21 | Ⅰ-Ⅲ | figure-of-8 coil | right DLPFC | 1Hz(80%) | C:Sham rTMS E:rTMS | 10(10 days) | UPDRS III,UPDRS,HAMD,MoCA | 12000 |
| Hamada M 2008 | Japan | C: 43 E: 55 | C: 67.4 ± 8.5 E: 65.3 ± 8.9 | Ⅱ-Ⅲ | figure-of-8 coil | SMA | 5 Hz(110%) | C:Sham rTMS E:rTMS | 8(8 weeks) | UPDRS-III,UPDRS,HAMD | 8000 |
| Bagattini C 2020 | Italy | C: 23/11 E: 27/10 | C: 73.35 ± 1.09 E: 73.56 ± 4.91 | C: 7.91 ± 0.67 E: 8.85 ± 3.91 | figure-of-8 coil | left DLPFC | 20Hz(100%) | C:Sham rTMS+CT E:rTMS+CT | 20(4 weeks) | MMSE,GDS | 40000 |
| Chen HF 2023 | China | C:6/2 E:18/11 | C:67.17 ± 8.75 E:66.67 ± 7.48 | C:11.33 ± 3.62 E:10.97 ± 4.08 | NR | left angular gyrus | 20Hz(100%) | C:Sham rTMS E:rTMS | 20(4 weeks) | MMSE,MoCA-BJ | 32000 |
| Cotelli M 2010 | Italy | C:5 E:5 | C:74.4 ± 63.8 E:71.2 ± 6.1 | C:4.8 ± 0.4 E:6.4 ± 1.3 | NR | DLPFC | 20Hz(100%) | C:Sham rTMS+Real rTMS E:rTMS | 20(4 weeks) | MMSE | C:20000 E:40000 |
| Hoy KE 2023 | Australia | C:29/20 E:27/18 | C:75.89 ± 6.89 E:75.03 ± 7.09 | C:13.00 ± 2.55 E:12.73 ± 3.50 | figure-of-8 coil | right and left DLPFC,PPC | 5Hz(100%) | C:Sham iTBS E:iTBS | 21 | ADAS-Cog,GDS | 50400 |
| Jia Y 2021 | China | C:34/23 E:35/25 | C:73.41 ± 7.73 E:71.41 ± 8.85 | C:7.50 ± 5.19 E:7.70 ± 5.26 | figure-of-8 coil | left parietal cortex | 10Hz(100-110%) | C:Sham rTMS E:rTMS | 10(2 weeks) | MMSE,CDR, | 8000 |
| Jung YH 2024 | Korea | C:12/6 E:18/12 | C:69.9±7.1 E:69.8±9.1 | 四C:16 (12.0-17.5) E:16 (12.8-16) | NR | left parietal area | 20Hz(100%) | C:Sham rTMS E:rTMS | 20(4 weeks) | ADAS-Cog,MMSE,MOCA,CDR-SOB | 32000 |
| Lee J 2016 | Korea | C: 8/5 E: 18/10 | C: 70.3±4.8 E: 72.1±7.6 | C: 9.9 ± 3.7 E: 9.9 ± 4.8 | NR | left DLPFC and both pSAC  areas | 10Hz(90%) | C:Sham rTMS+CT E:rTMS | 30(6 weeks) | ADAS-Cog,MMSE,GDS | 36000 |
| Leocani L 2021 | Italy | C: 6/3 E: 7/3 | C: 72.6 ± 8.3 E: 69.6 ± 7.9 | C:7.8 ±3.4 E: 9.2 ± 4.5 | H2-coil | right and left prefrontal cortices and in temporal-parietal areas | 10Hz(120%) | C:Sham rTMS E:rTMS | 16(8 weeks) | ADAS-Cog | 13440 |
| Li X 2021 | China | C:37/17 E:38/14 | C:64.58 ± 7.88 E:65.97 ± 8.47 | C:6.75±4.51 E:5.65±3.21 | figure-of-8 coil | left DLPFC | 20Hz(100%) | C:Sham rTMS E:rTMS | 30(6 weeks) | MMSE,ADAS-Cog | 60000 |
| Vecchio F 2022 | Italy | C: 17/7 E: 30/16 | C: 72.24 ±2.29 E: 71.07 ±1.25 | C: 11.47 ±1.23 E: 13.87 ±0.78 | figure-of-8 coil | Broca’s area, right and left DLPFC,  Wernicke’s area,right and left pSAC | 10Hz(90-110%) | C:Sham rTMS+CT E:rTMS+CT | 30(6 weeks) | ADAS-Cog | 42000 |
| Wei L 2022 | China | C:27/20 E:29/20 | C:71.67 ± 7.16 E:70.00 ± 8.63 | C:6.63 ± 4.99 E:7.34 ± 5.54 | figure-of-8 coil | left lateral parietal | 10Hz(100-110%) | C:Sham rTMS E:rTMS | 10(2 weeks) | MMSE,CDR | 8000 |
| Wu X 2022 | China | C:24/14 E:23/12 | C:66.35±7.99 E:66.46±8.25 | C:9.52±4.08 E:9.71±4.65 | figure-of-8 coil | left DLPFC | 5Hz(70%) | C:Sham iTBS E:iTBS | 14（2 weeks） | MMSE,MoCA | 25200 |
| Wu Y, 2015 | China | C:26/15 E:26/16 | C:71.9±4.8 E:71.4±4.9 | C:11.5±2.1 E:11.4±2.7 | figure-of-8 coil | left DLPFC | 20Hz(80%) | C:Sham rTMS E:rTMS | 20(4 weeks) | ADAS-Cog | 24000 |
| Yao Q, 2022 | China | C:12/6 E:15/7 | C:67.60 ± 7.88 E: 63.87± 6.85 | C:9.40±3.63 E:10.53±3.8 | figure-of-8 coil | bilateral cere bellum crus II | 5Hz(90%) | C:Sham rTMS E:rTMS | 20(4 weeks) | MMSE,ADAS-Cog,MoCA | 24000 |
| Zhao J, 2017 | China | C:13/7 E:17/10 | C:71.4±5.2 E:69.3±5.8 | C:4.9±3.5 E: 4.8±1.9 | NR | parietal P3/P4 and posterior  temporal T5/T6 | 20Hz | C:Sham rTMS E:rTMS | 30(6 weeks) | MMSE,ADAS-Cog,MoCA | 42000 |
| Zhou X 2022 | China | C:32/22 E:33/22 | C:74 ± 11.67  E:70 ± 8.89 | C:11±4.26  E:11±4.07 | NR | left DLPFC | 10Hz(120%) | C:Sham rTMS E:rTMS | 20(4 weeks) | ADAS-Cog | 30000 |
| Koch G,2022 | Italy | C: 25/12 E: 25/14 | C: 72.3 ± 7.2 E: 75.0 ± 5.6 | C: 8.6 ± 4.1 E: 10.2 ± 4.4 | figure-of-8 coil | precuneus | 20Hz | C:Sham rTMS E:rTMS | 32(24 weeks) | ADAS-Cog,MMSE,CDR | 51200 |
| Padala PR 2020 | USA | C: 11/1 E: 9/8 | C: 79.6 ± 7.7 E: 74.3 ±5.7 | college degree C: 3 E: 7 | NR | left DLPFC | 20Hz(120%) | C:Sham rTMS E:rTMS | 20(4 weeks) | MMSE | 60000 |
| Saitoh Y 2022 | Japan | C: 12/8 E: 15/7 | C: 75.8 E: 76.2 | C: 13.2 E: 13.7 | figure-of-8 coil | bilateral DLPFC | 10Hz(120%) | C:Sham rTMS E:rTMS | 10(4 weeks) | MMSE, ADAS-Cog,MoCA | 12000 |
| Bagattini C 2020 | Italy | C: 23/11 E: 27/10 | C: 73.35 ± 1.09 E: 73.56 ± 4.91 | C: 7.91 ± 0.67 E: 8.85 ± 3.91 | figure-of-8 coil | left DLPFC | 20Hz(100%) | C:Sham rTMS+CT E:rTMS+CT | 20(4 weeks) | MMSE,GDS | 40000 |
| Chen HF 2023 | China | C:6/2 E:18/11 | C:67.17 ± 8.75 E:66.67 ± 7.48 | C:11.33 ± 3.62 E:10.97 ± 4.08 | NR | left angular gyrus | 20Hz(100%) | C:Sham rTMS E:rTMS | 20(4 weeks) | MMSE,MoCA-BJ | 32000 |
| Cotelli M 2010 | Italy | C:5 E:5 | C:74.4 ± 63.8 E:71.2 ± 6.1 | C:4.8 ± 0.4 E:6.4 ± 1.3 | NR | DLPFC | 20Hz(100%) | C:Sham rTMS+Real rTMS E:rTMS | 20(4 weeks) | MMSE | C:20000 E:40000 |

**Abbreviation:** Y, Year; F, Female; E, Experimental group; C, Control group; NR, Not reported; HY, Hoehn and Yahr scale; TMS, Repetitive transcranial magnetic stimulation; iTBS, intermittent Theta Burst Stimulation;CTBS, continuous theta-burst stimulation; CT, cognitive behavior therapy; DLPFC, The dorsolateral prefrontal cortex; M1, Motor cortex; PFC,Prefrontal Cortex; SMA, supplementary motor area; pSAC, parietal superior association cortex; EDB,extensor digitorum brevis; PPC, Posterior Parietal Cortex; UPDRS Ⅲ,Unified Parkinson’s Disease Rating Scale Part Three; FOGQ, Freezing of Gait Questionnaire; TUG,Timed Up and Go test; MMSE, Minimum Mental State Examination; HAMD, Hamilton Depression Scale; MoCA, Montreal Cognitive Assessment; HAMA, Hamilton Anxiety Scale; ADAS-Cog: Alzheimer’s Disease Assessment Scale-Cognition; CDR, Clinical Dementia Rating; GDS, Geriatric Depression Scale.

**eTable 4. Grade Quality of evidence**

| **Quality assessment** | | | | | | | **No of patients** | | **Effect** | | **Quality** | **Importance** |  |
| --- | --- | --- | --- | --- | --- | --- | --- | --- | --- | --- | --- | --- | --- |
| **No of studies** | **Design** | **Risk of bias** | **Inconsistency** | **Indirectness** | **Imprecision** | **Other considerations** | **intervention** | **Control** | **Relative (95% CI)** | **Absolute** |  |  |  |
| **New Outcome (Better indicated by lower values)** | | | | | | | | | | | | |  |
| 13 | randomised trials | no serious risk of bias | no serious inconsistency | no serious indirectness | no serious imprecision | none | 287 | 265 | - | SMD 0.91 lower (1.98 to 0.17 lower) | ⊕⊕⊕⊕ HIGH |  | UPDRS |
| 24 | randomised trials | serious^1^ | no serious inconsistency | no serious indirectness | no serious imprecision | none | 464 | 427 | - | SMD 0.66 lower (0.91 to 0.41 lower) | ⊕⊕⊕O MODERATE |  | UPDRSⅢ |
| 7 | randomised trials | serious^1^ | no serious inconsistency | no serious indirectness | no serious imprecision | none | 145 | 133 | - | SMD 1.40 lower (2.71 to 0.10 lower) | ⊕⊕⊕O MODERATE |  | HAMD |
| 10 | randomised trials | serious^1^ | no serious inconsistency | no serious indirectness | no serious imprecision | none | 203 | 195 | - | SMD 0.41 lower (0.61 to 0.21 lower) | ⊕⊕⊕O MODERATE |  | TUG |
| 6 | randomised trials | serious^1^ | no serious inconsistency | no serious indirectness | no serious imprecision | none | 90 | 86 | - | SMD 0.62 lower (0.92 to 0.31 lower) | ⊕⊕⊕O MODERATE |  | FOGQ |
| 7 | randomised trials | serious^1^ | no serious inconsistency | no serious indirectness | no serious imprecision | none | 109 | 105 | - | SMD 0.61 lower (0.88 to 0.33 lower) | ⊕⊕⊕O MODERATE |  | BDI |
| 5 | randomised trials | serious^1^ | serious^2^ | no serious indirectness | no serious imprecision | none | 82 | 73 | - | SMD 0.26 higher (0.06 lower to 0.58 higher) | ⊕⊕OO LOW |  | MMSE |
| 4 | randomised trials | no serious risk of bias | serious^1^ | no serious indirectness | no serious imprecision | none | 96 | 96 | - | SMD 0.78 lower (1.97 lower to 0.40 higher) | ⊕⊕⊕O MODERATE |  | HAMA |
| 3 | randomised trials | no serious risk of bias | serious^1^ | no serious indirectness | no serious imprecision | none | 76 | 64 | - | SMD 0.12 lower (1.13 lower to 0.89 higher) | ⊕⊕⊕O MODERATE |  | MoCA |
| 12 | randomised trials | serious^1^ | no serious inconsistency | no serious indirectness | no serious imprecision | none | 269 | 229 | - | SMD 0.2 lower (0.38 to 0.02 lower) | ⊕⊕⊕O MODERATE |  | ADAS-Cog |
| 14 | randomised trials | no serious risk of bias | no serious inconsistency | no serious indirectness | no serious imprecision | none | 292 | 249 | - | SMD 0.43 higher (0.01 to 0.84 higher) | ⊕⊕⊕⊕ HIGH |  | MMSE |
| 6 | randomised trials | no serious risk of bias | no serious inconsistency | no serious indirectness | no serious imprecision | none | 106 | 79 | - | SMD 0.38 higher (0.08 to 0.67 higher) | ⊕⊕⊕⊕ HIGH |  | MoCA |
| 4 | randomised trials | no serious risk of bias | serious^1^ | no serious indirectness | no serious imprecision | none | 107 | 98 | - | SMD 0.03 lower (0.3 lower to 0.25 higher) | ⊕⊕⊕O MODERATE |  | CDR |
| 3 | randomised trials | serious^1^ | no serious inconsistency | no serious indirectness | no serious imprecision | none | 74 | 58 | - | SMD 0.06 higher (0.28 lower to 0.41 higher) | ⊕⊕⊕O MODERATE |  | GDS |

**Other forest plot results**

**1.eFigure 1: Forest plot of ≤1 Hz**

**
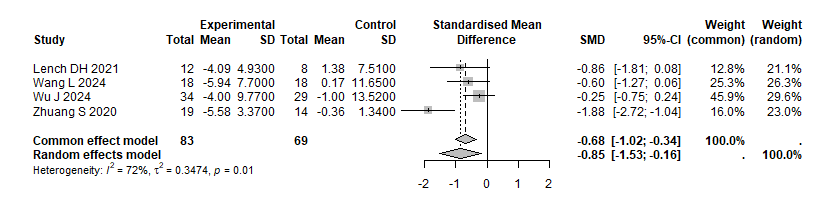
**

**2.eFigure 2: Forest plot of 1-10Hz**

**
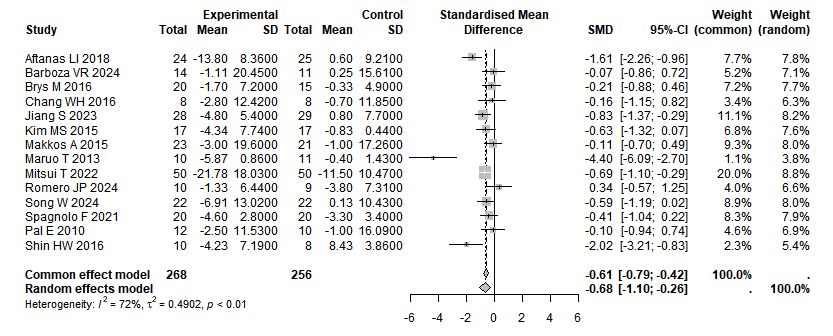
**

**3.eFigure 3: Forest plot of >10Hz**

**
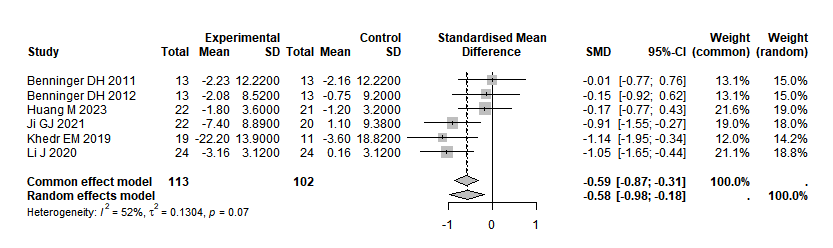
**

**4.eFigure 4: Forest plot of Single Target Point**

**
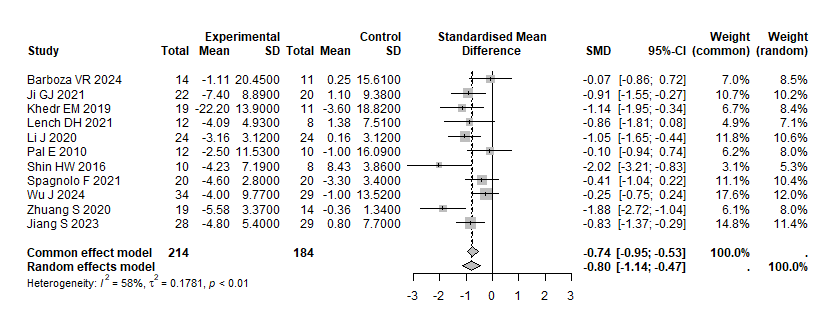
**

**5.eFigure 5: Forest plot of Multiple Target Points**

**
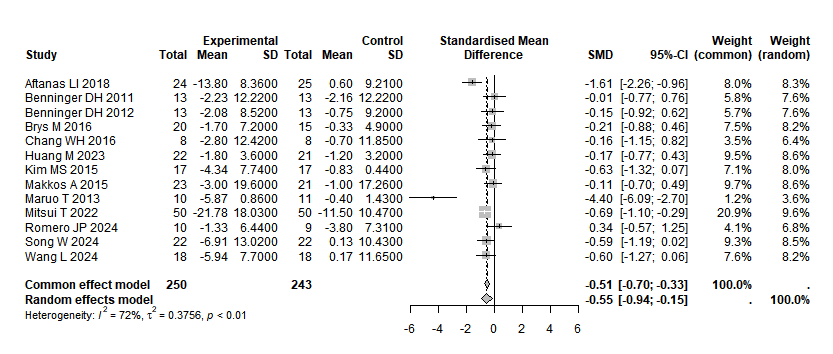
**

1. **eFigure 6: Forest plot of UPDRS total score.**

**
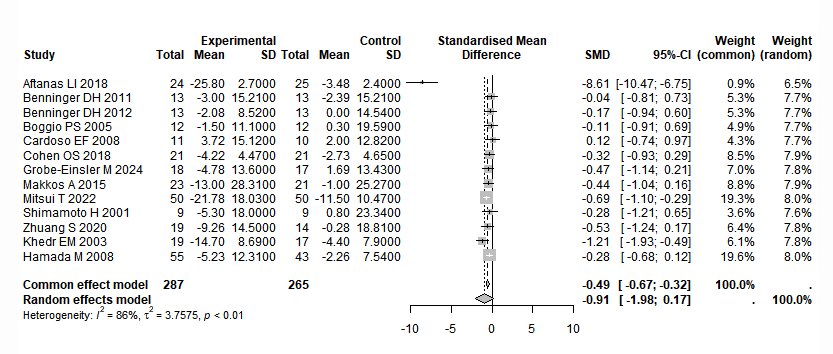
**

1. **eFigure 7: Forest plot of TUG.**

**
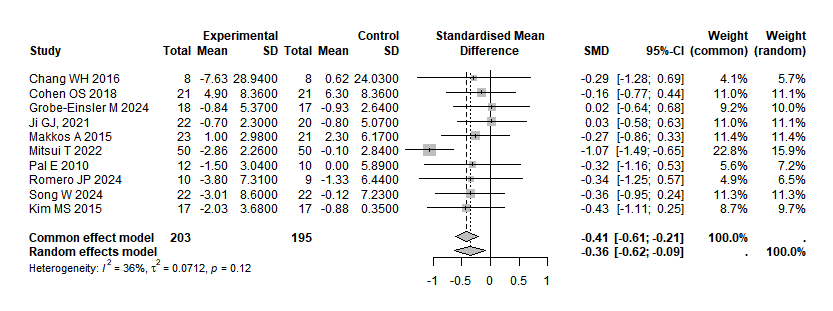
**

**8.eFigure 8: Forest plot of FOGQ.**

**
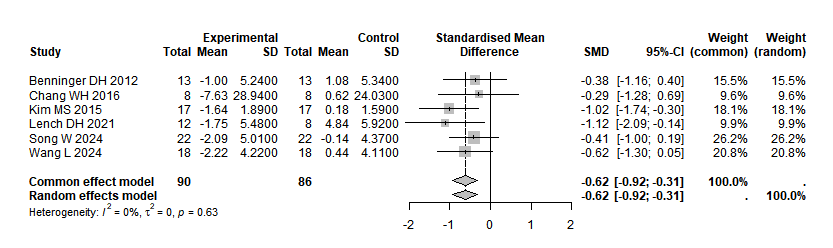
**

**9.eFigure 9: Forest plot of HAMD.**

**
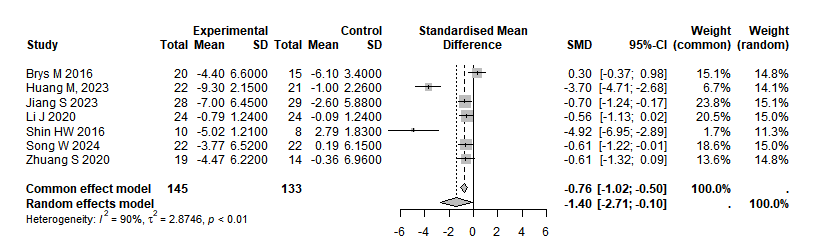
**

**10.eFigure 10: Forest plot of BDI.**

**
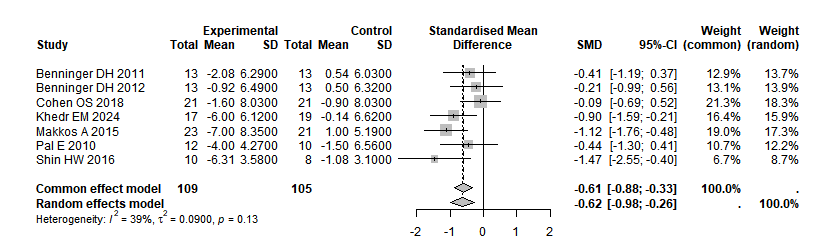
**

**11.eFigure 11: Forest plot of HAMA.**

**
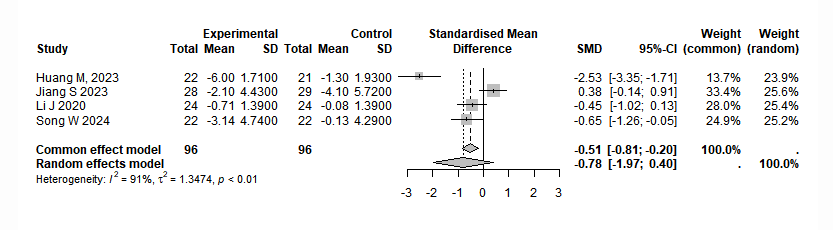
**

**12.eFigure 12: Forest plot of MoCA.**

**
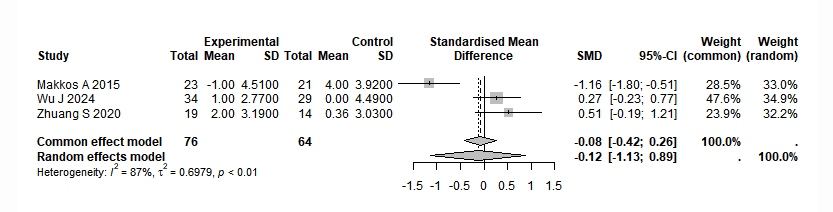
**

**13.eFigure 13: Forest plot of MMSE.**

**
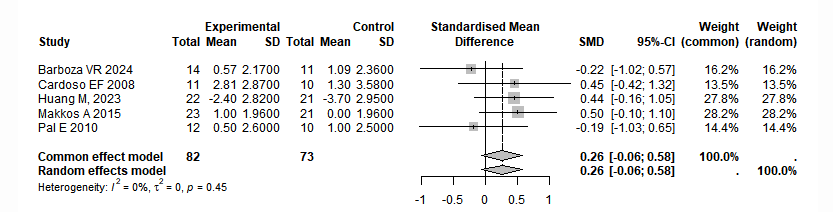
**

**14.eFigure 14: Forest plot of ≤10Hz.**

**
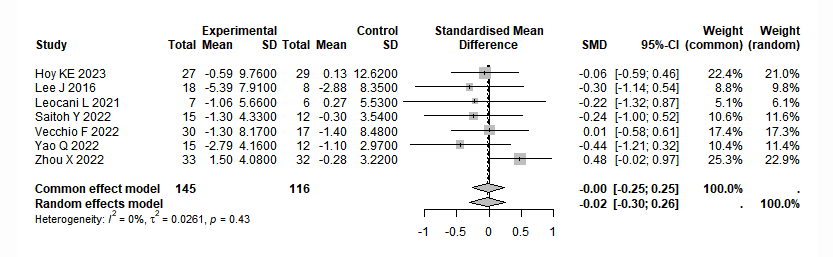
**

**15.eFigure 15: Forest plot of >10Hz.**

**
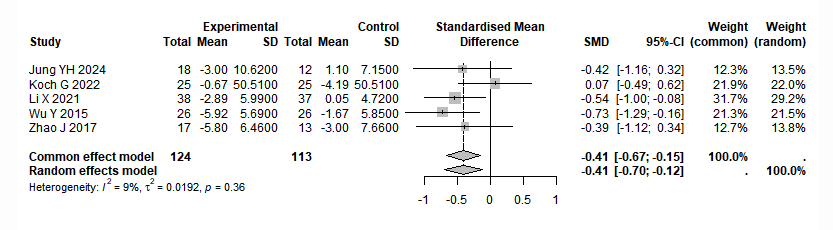
**

**16.eFigure 16: Forest plot of Single Target Point.**

**
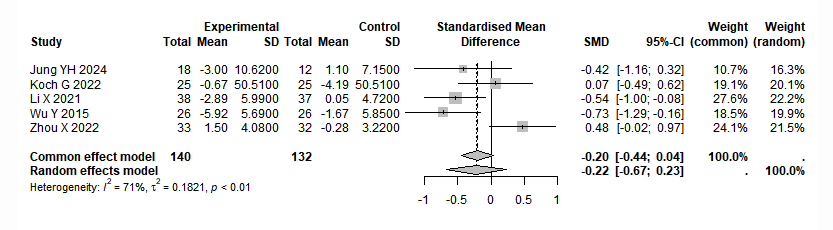
**

**17.eFigure 17: Forest plot of Multiple Target Points.**

**
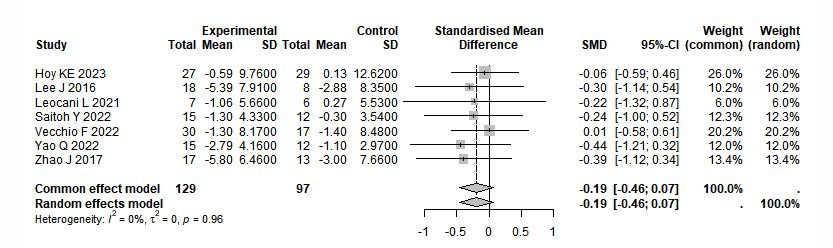
**

**18.eFigure 18: Forest plot of MMSE.**

**
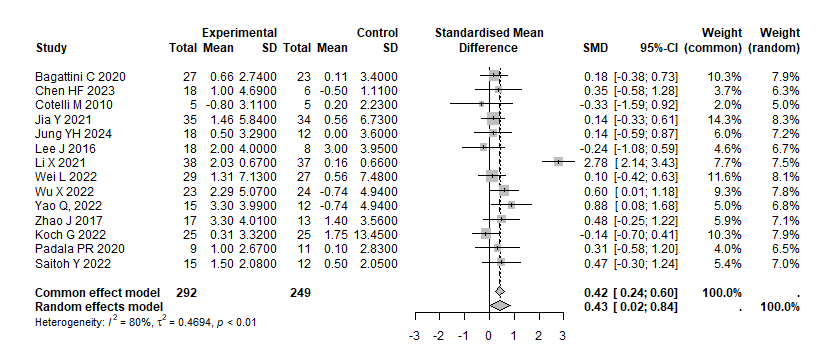
**

**19.eFigure 19: Forest plot of MoCA.**

**
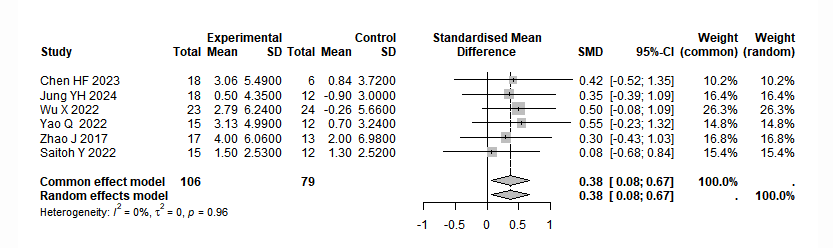
**

**20.eFigure 20: Forest plot of CDR.**

**
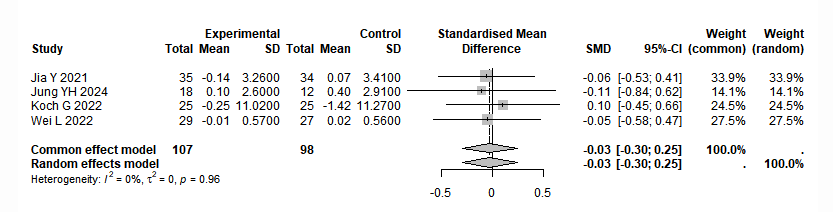
**

**21.eFigure 21: Forest plot of GDS.**

**
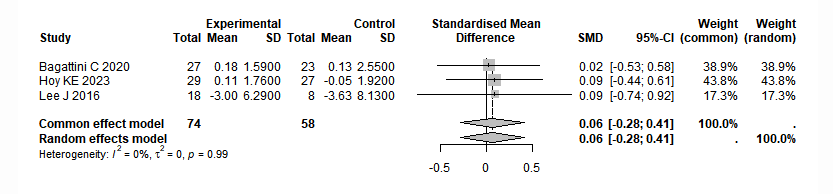
**

**Sensitivity Analysis**

**22.eFigure 22. shows the sensitivity analysis of UPDRS III.**

**
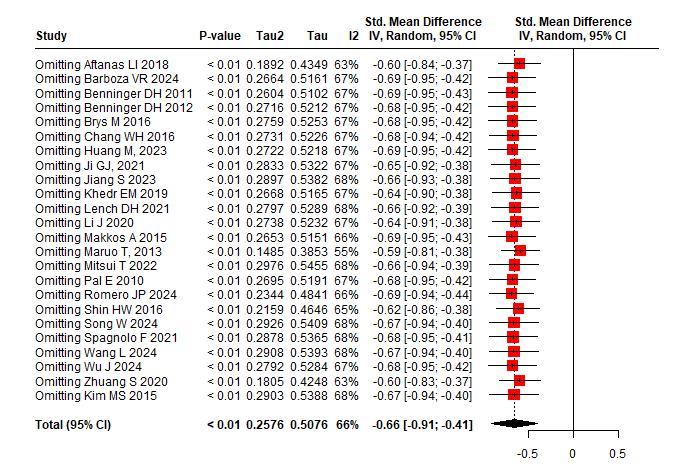
**

**23.eFigure 23. shows the sensitivity analysis of UPDRS total score.**

**
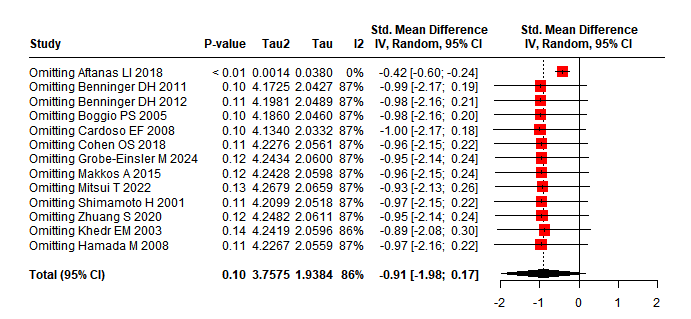
24.eFigure 24. shows the sensitivity analysis of TUG.**


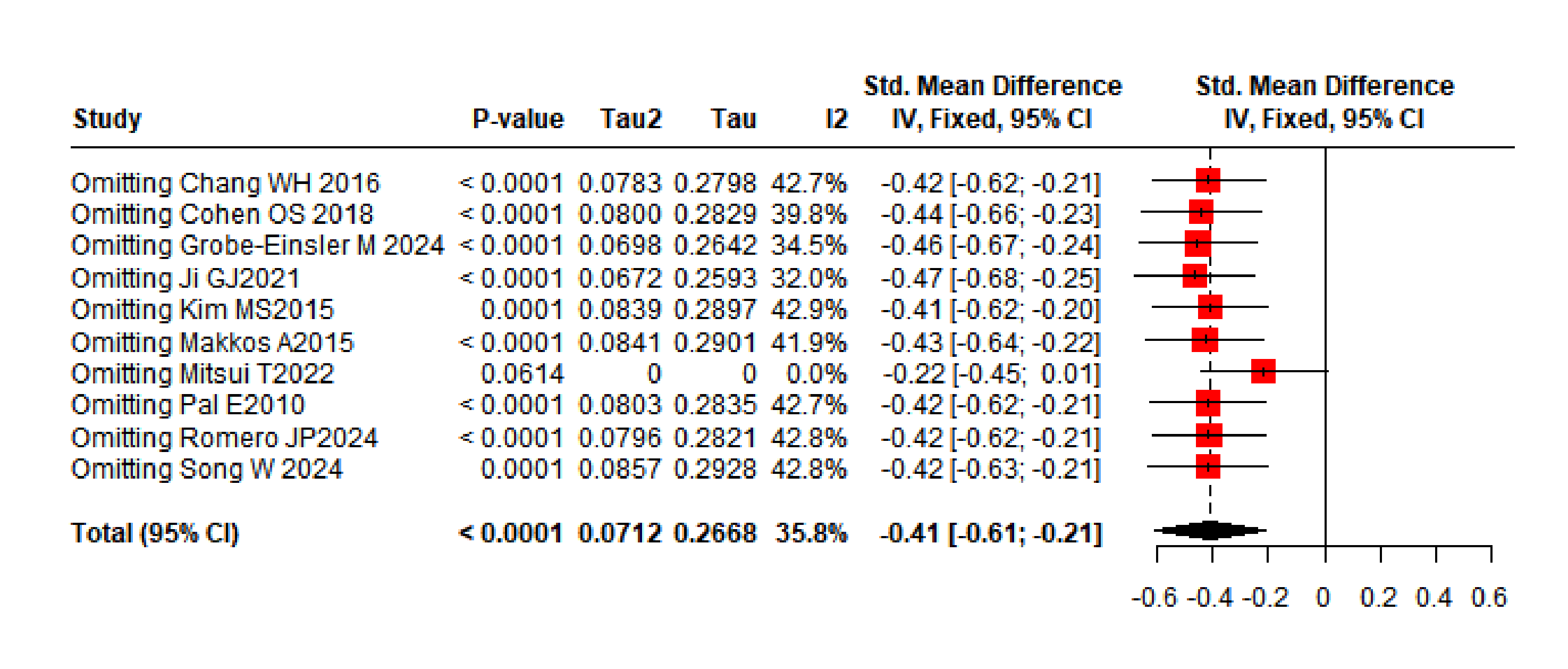


**25.eFigure 25. shows the sensitivity analysis of FOGQ.**

**
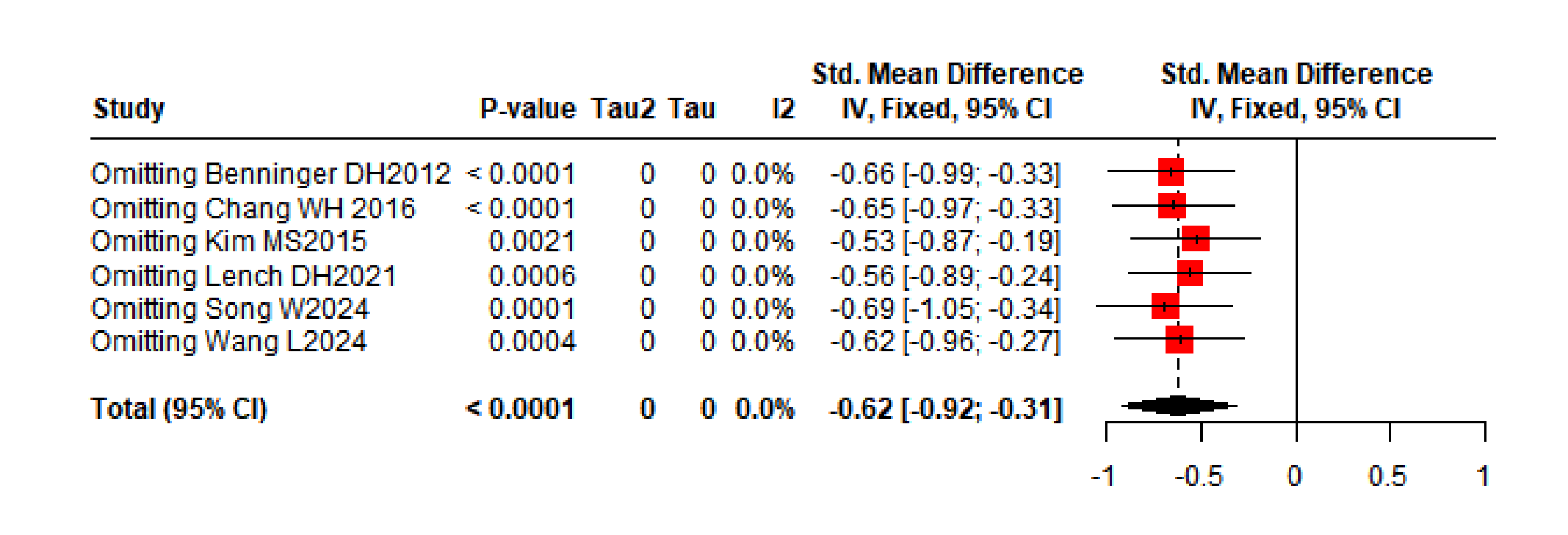
**

**26.eFigure 26. shows the sensitivity analysis of HAMD**

**
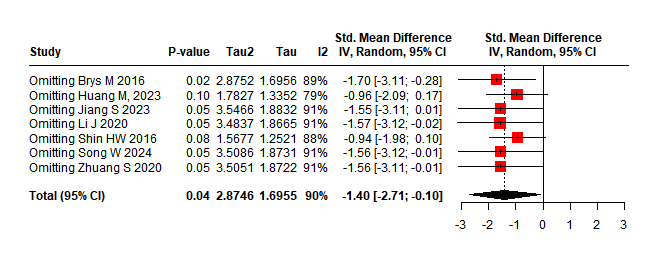
**

**27.eFigure 27. shows the sensitivity analysis of BDI.**

**
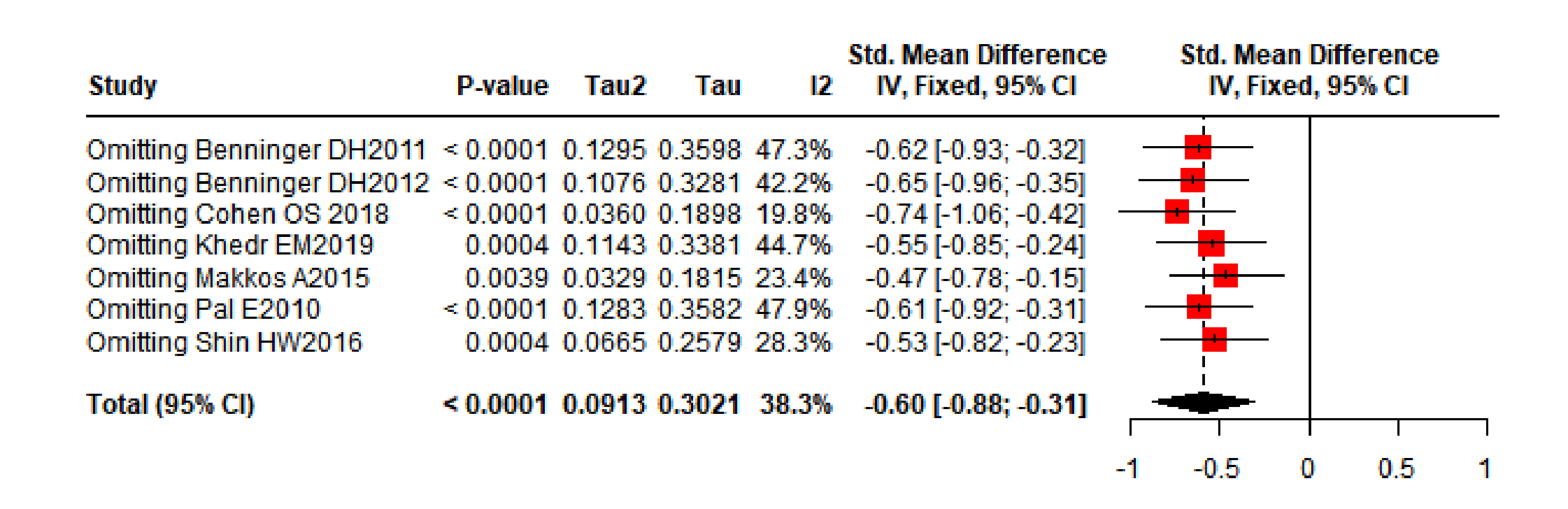
**

**28.eFigure 28. shows the sensitivity analysis of HAMA.**

**
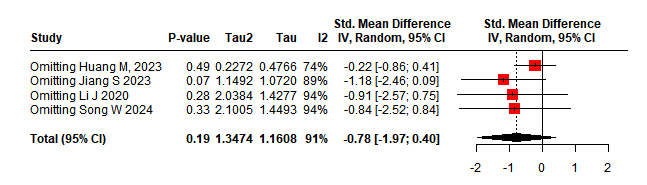
**

**29.eFigure 29. shows the sensitivity analysis of MoCA.**

**
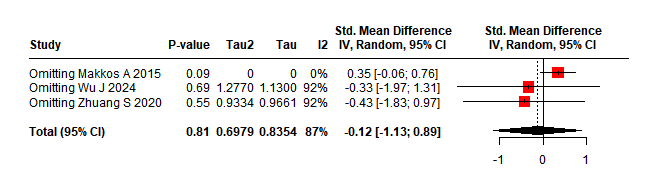
**

**30.eFigure 30. shows the sensitivity analysis of MMSE.**

**
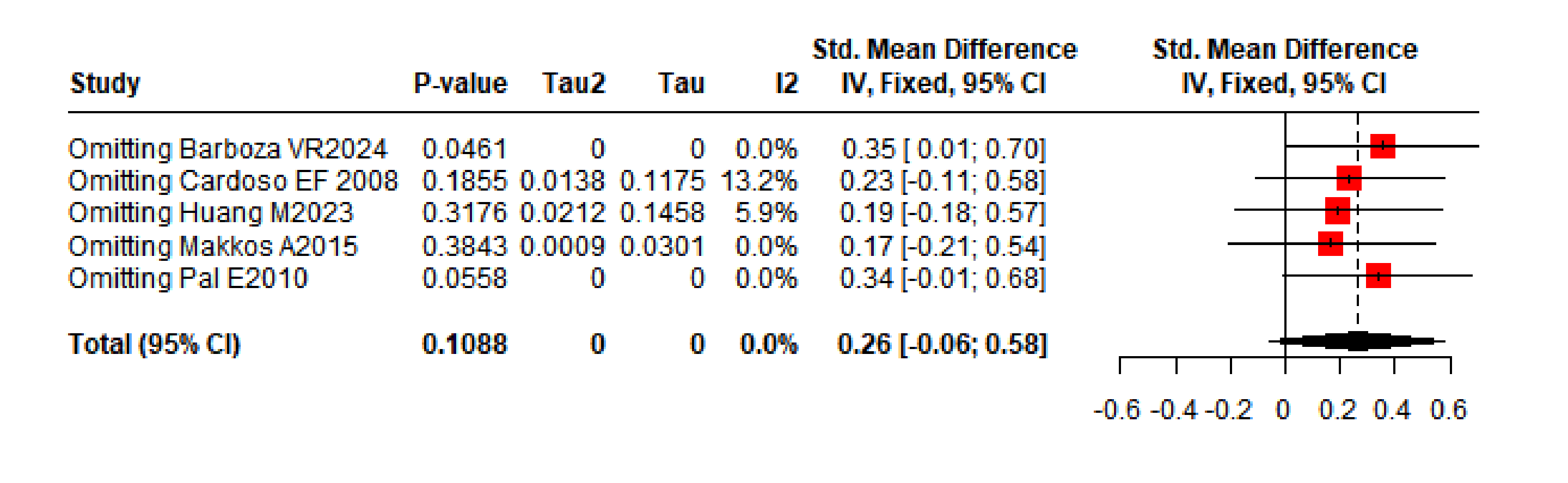
**

**31.eFigure 31. shows the sensitivity analysis of ADAS-Cog.**

**
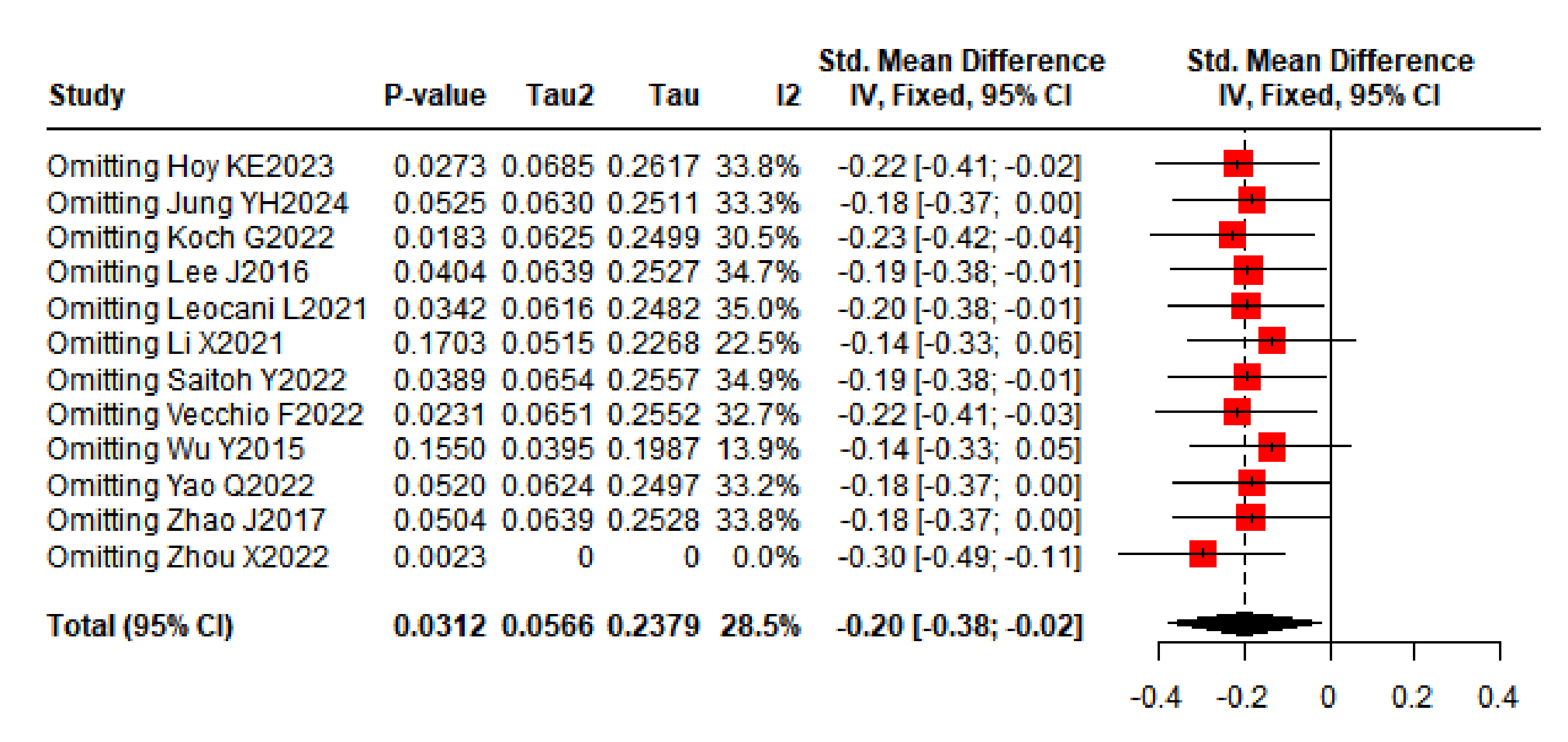
**

**32.eFigure 32. shows the sensitivity analysis of MMSE.**

**
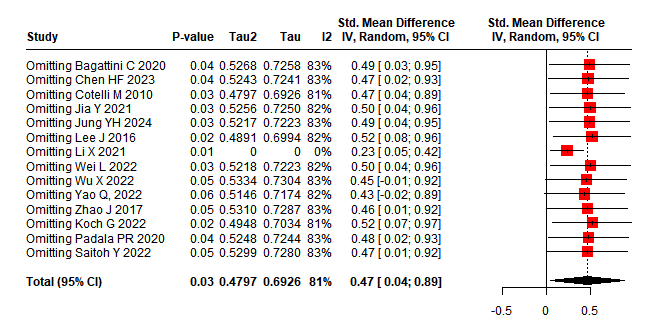
**

**33.eFigure 33. shows the sensitivity analysis of MoCA.**

**
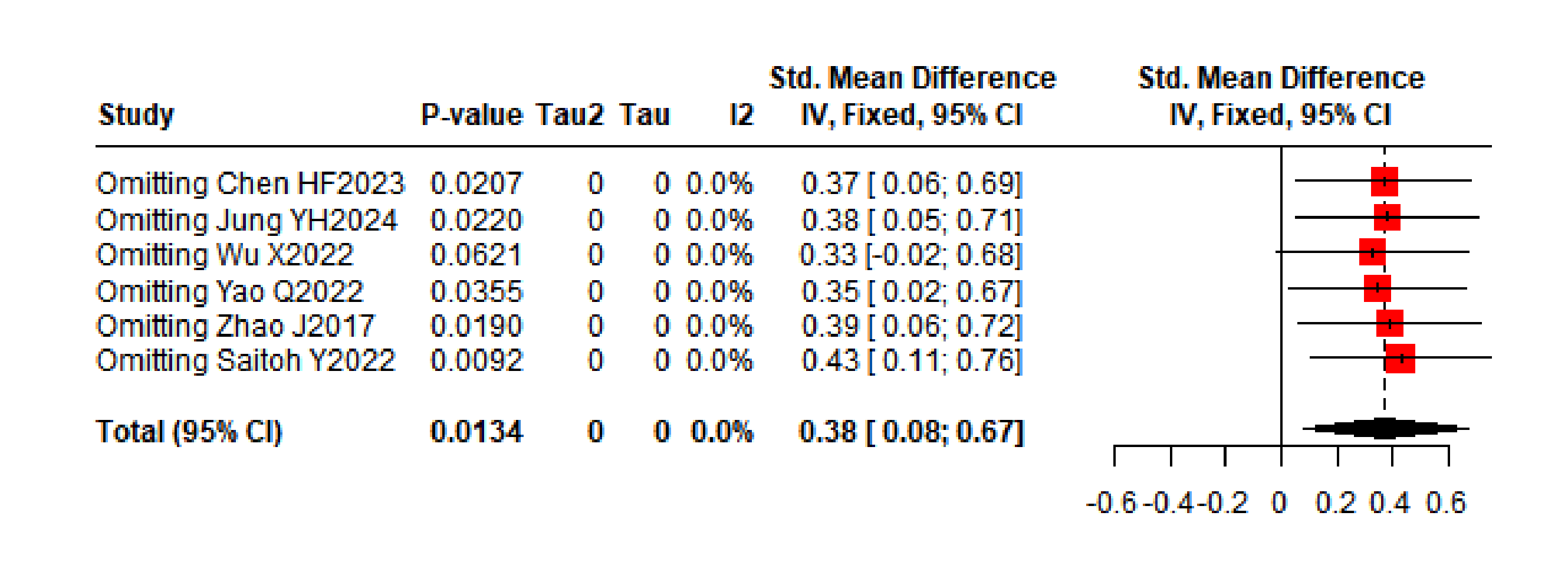
**

**34.eFigure 34. shows the sensitivity analysis of CDR.**

**
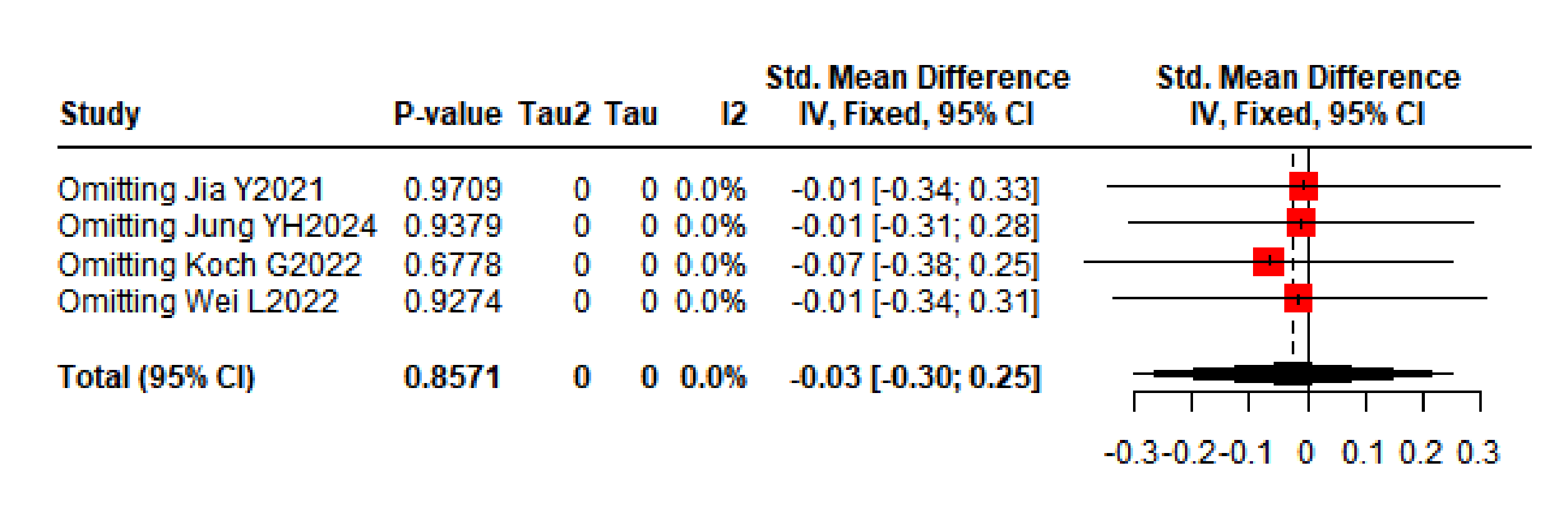
**

**35.eFigure 35. shows the sensitivity analysis of GDS.**

**
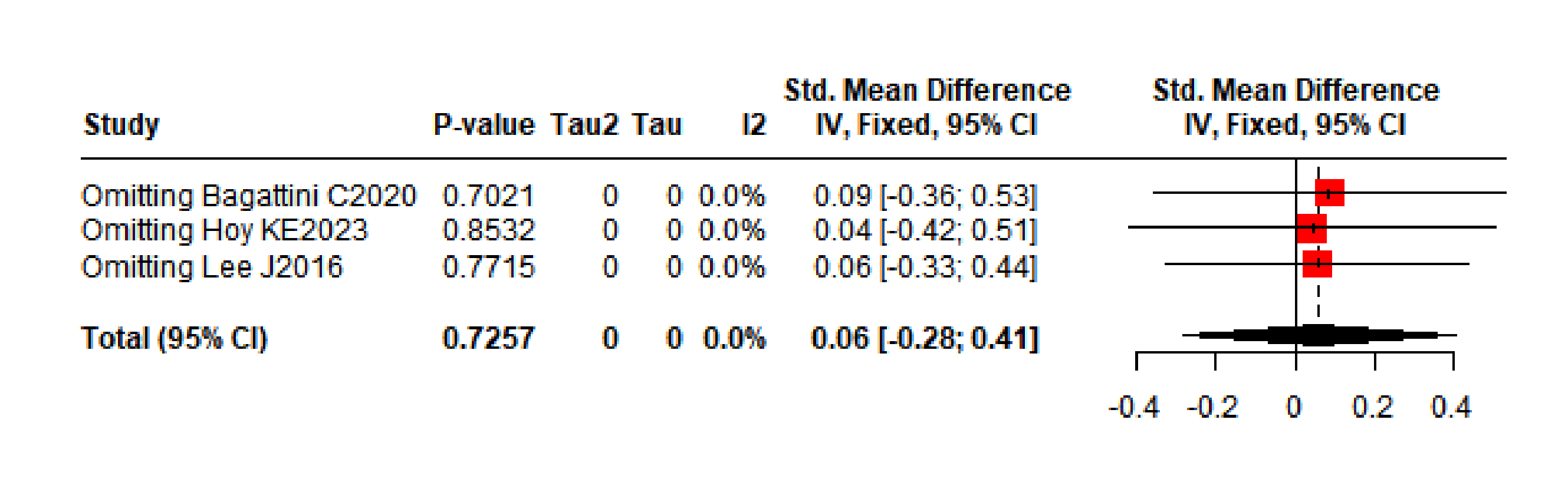
**

**Dose-response relationship**

**36.eFigure 36. Dose-response relationship between Total pulses and improvement of UPDRS III.(≤1Hz)**

**
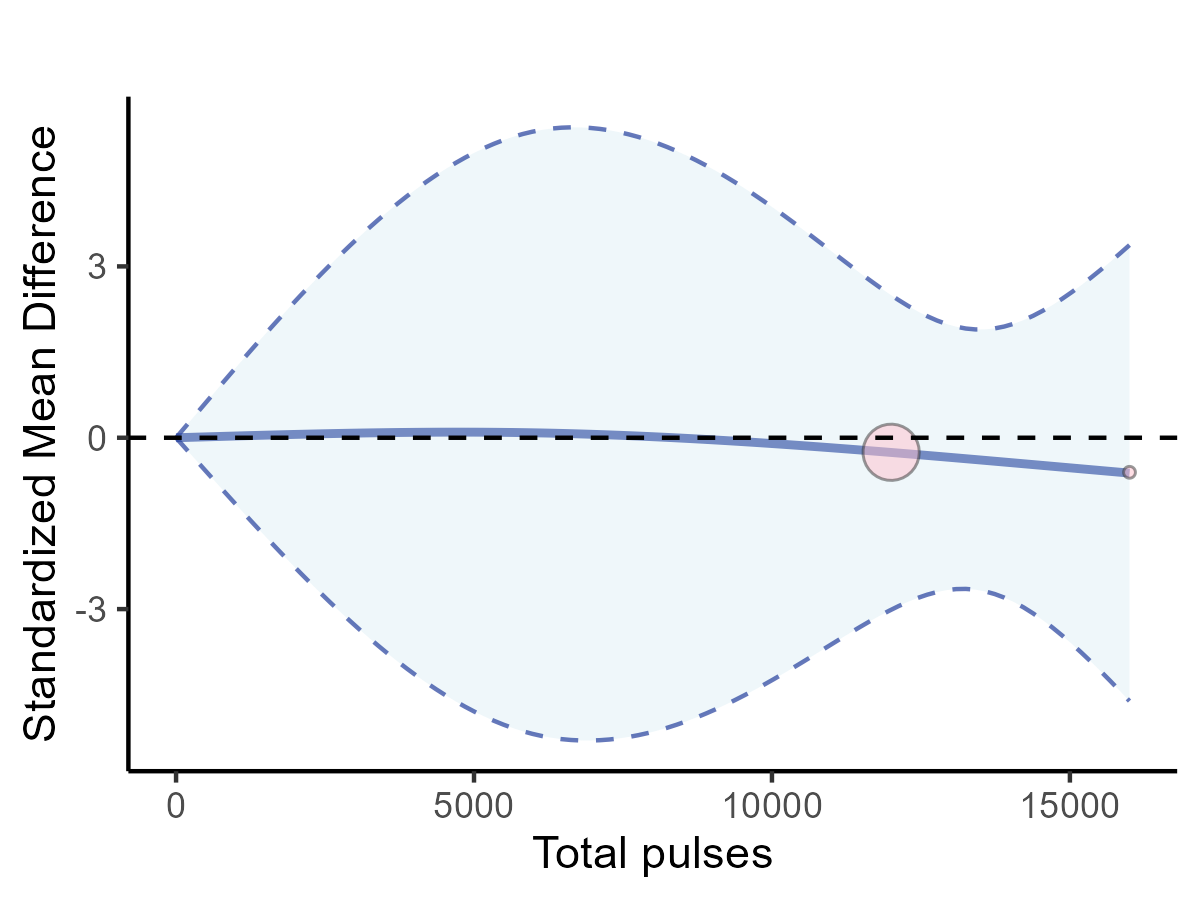
**

**37.eFigure 37. Dose-response relationship between Total pulses and improvement of UPDRS III.(1Hz-10Hz)**

**
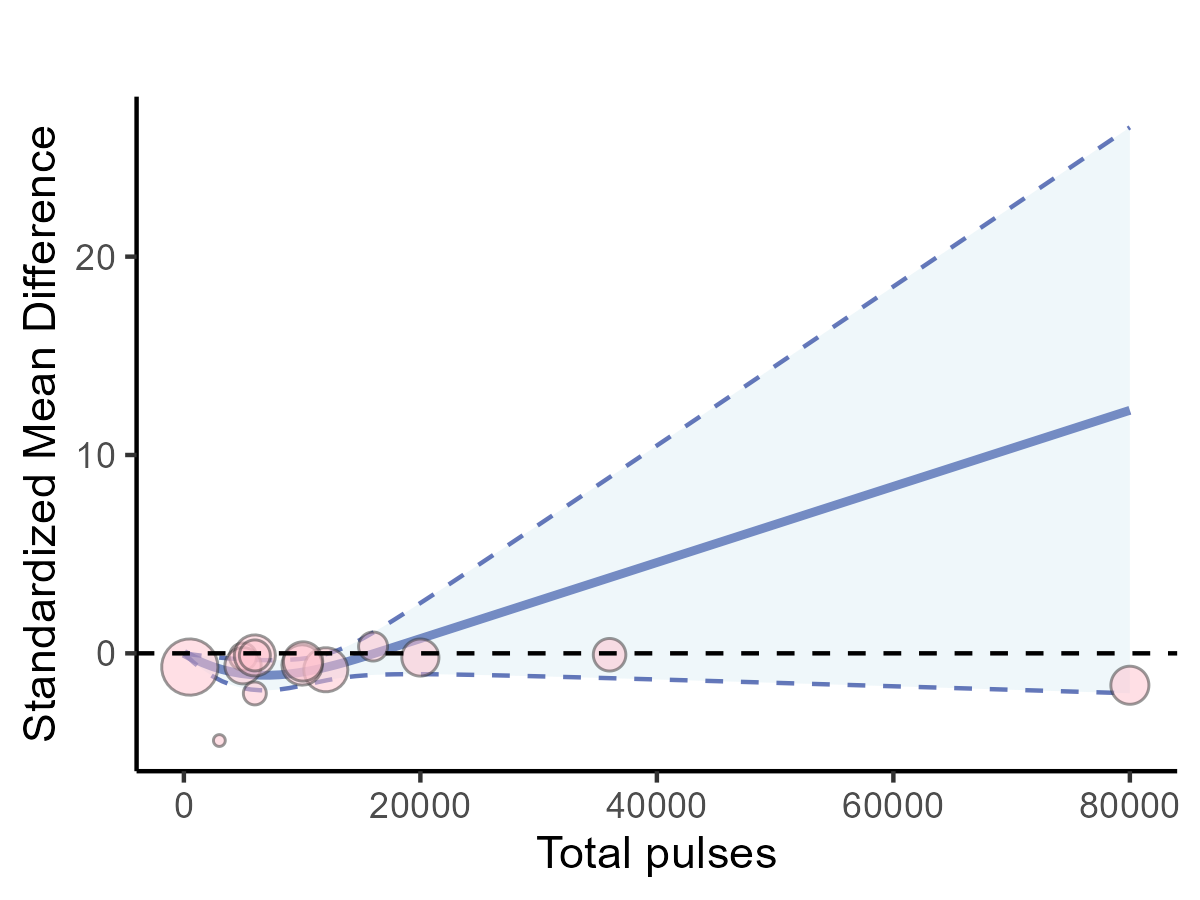
**

**38.eFigure 38. Dose-response relationship between Total pulses and improvement of UPDRS III.(>10Hz)**

**
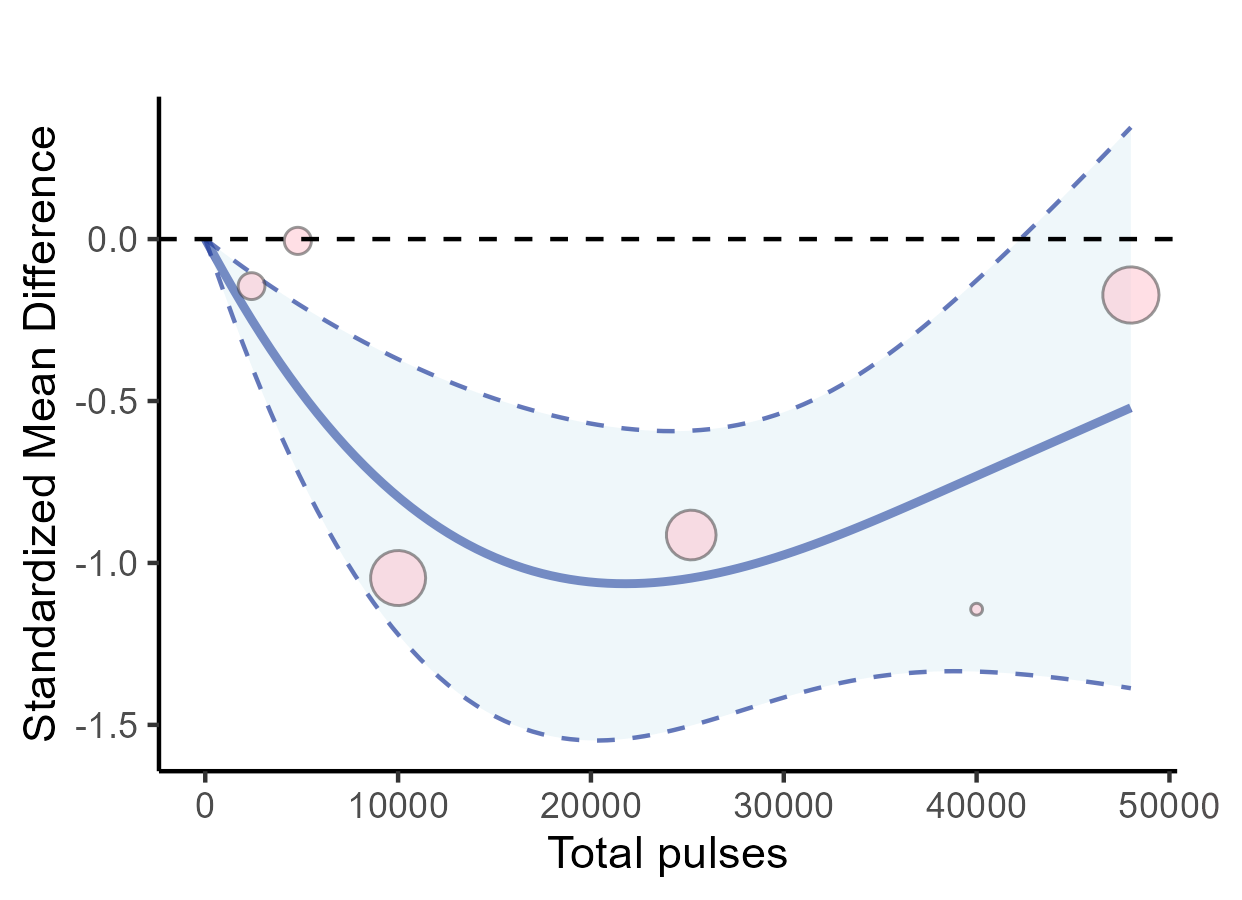
**

**39.eFigure 39. Dose-response relationship between Total pulses and improvement of UPDRS III.(Single Target Point)**

**
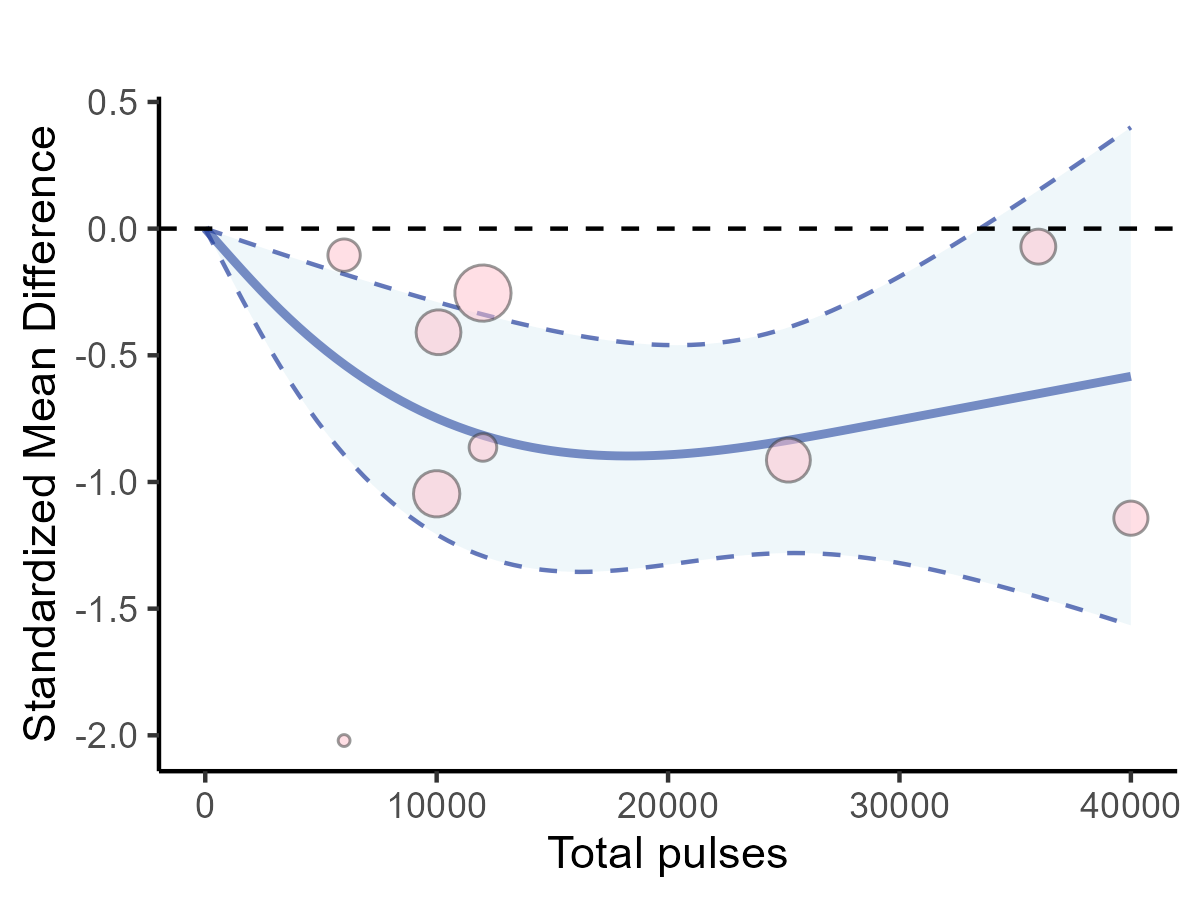
**

**40.eFigure 40. Dose-response relationship between Total pulses and improvement of UPDRS III.(Multiple Target Points)**

**
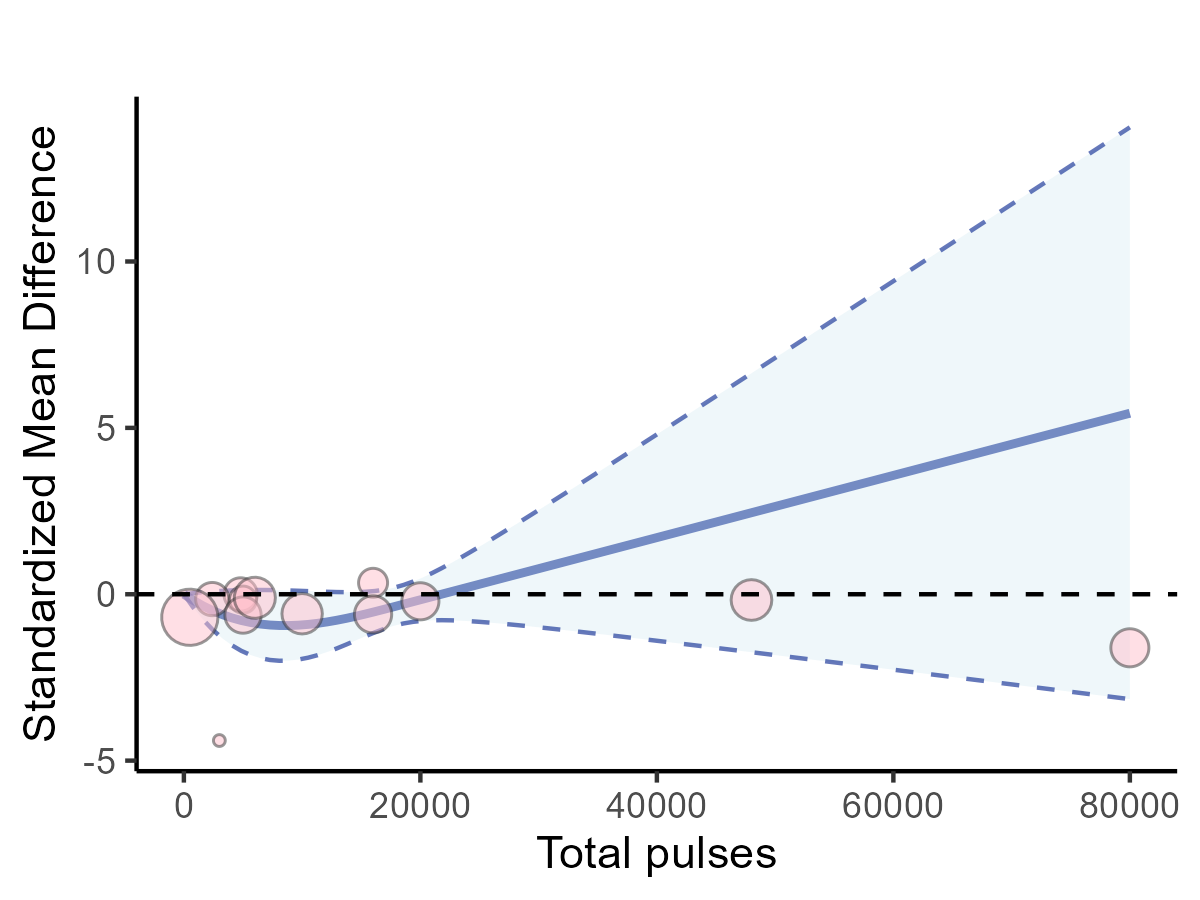
**

**41.eFigure 41. Dose-response relationship between Total pulses and improvement of ADAS-Cog.(≤ 10 Hz)**

**
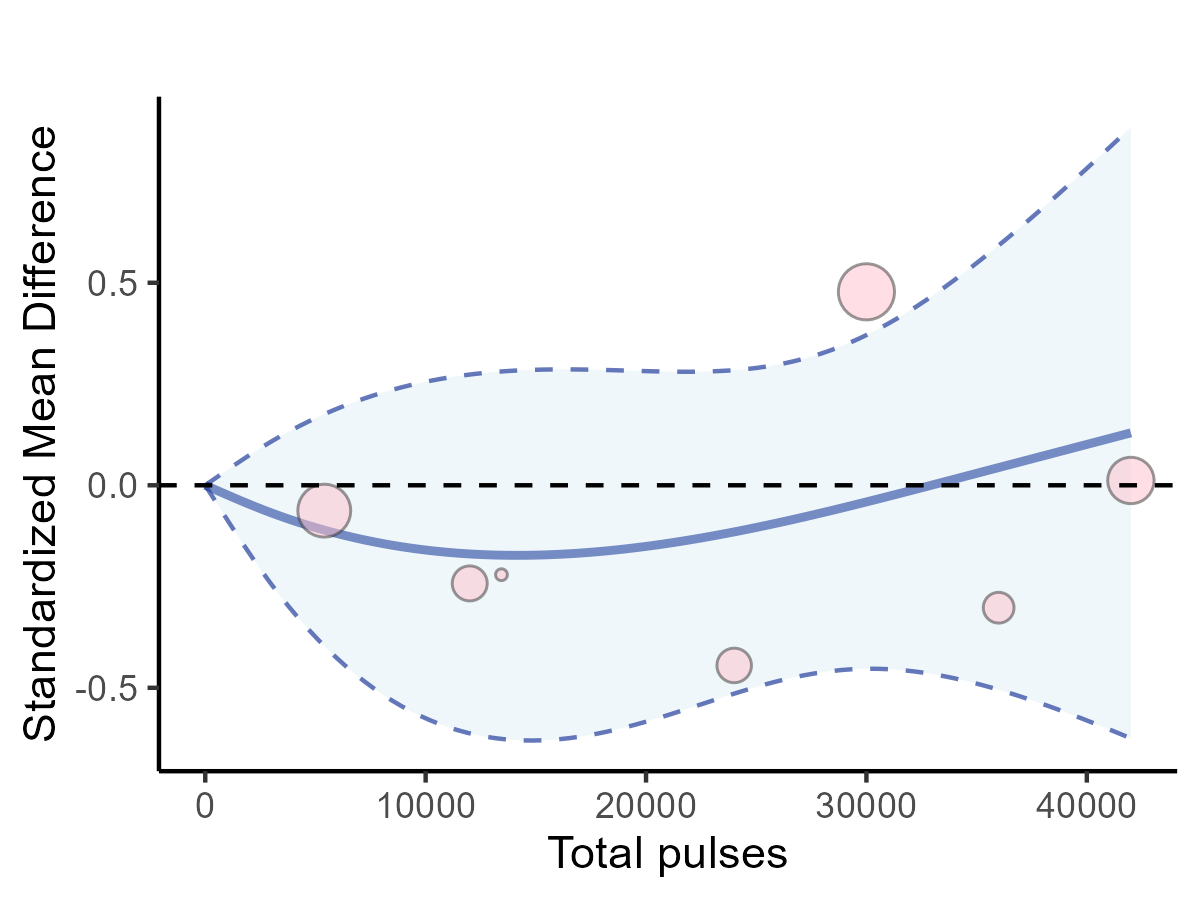
**

**42.eFigure 42. Dose-response relationship between Total pulses and improvement of ADAS-Cog.(> 10 Hz)**

**
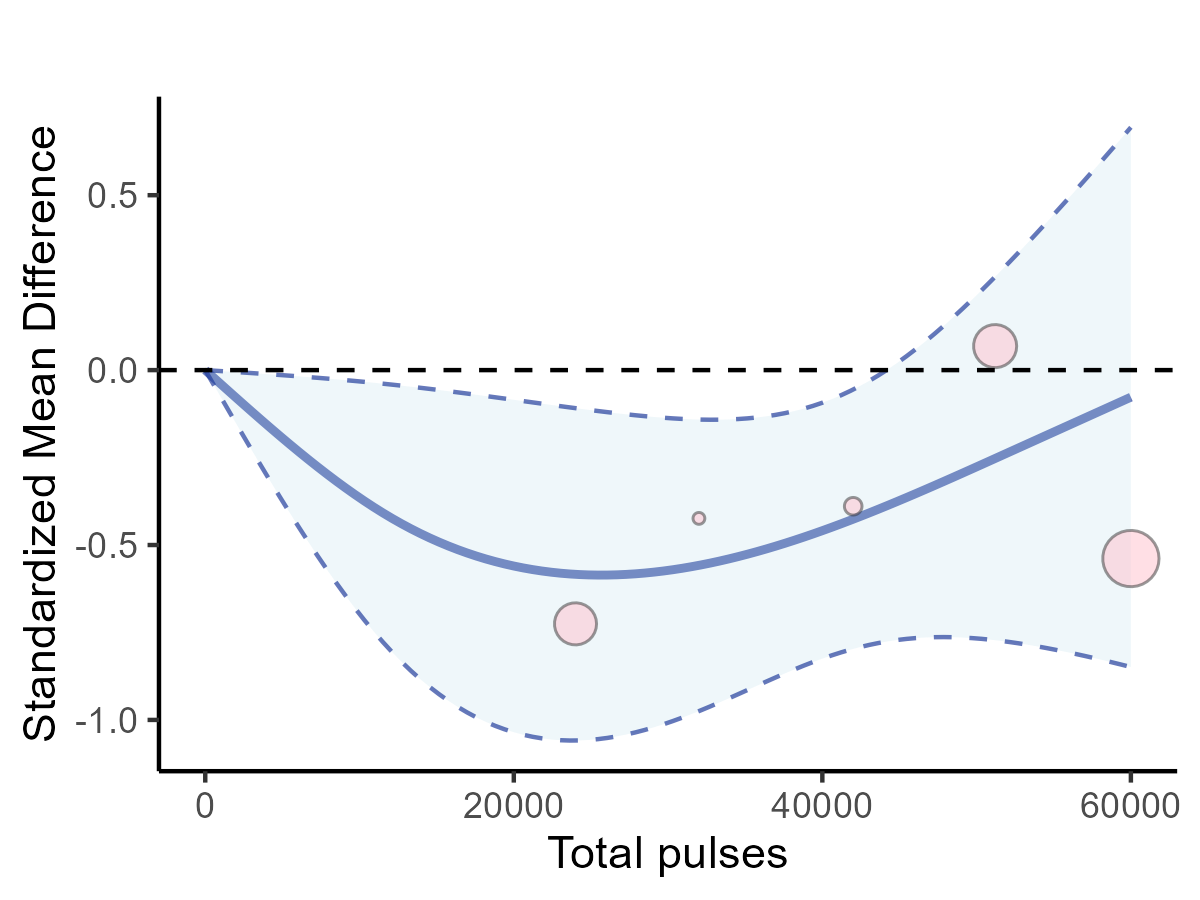
**

**43.eFigure 43. Dose-response relationship between Total pulses and improvement of ADAS-Cog.(Single Target Points)**

**
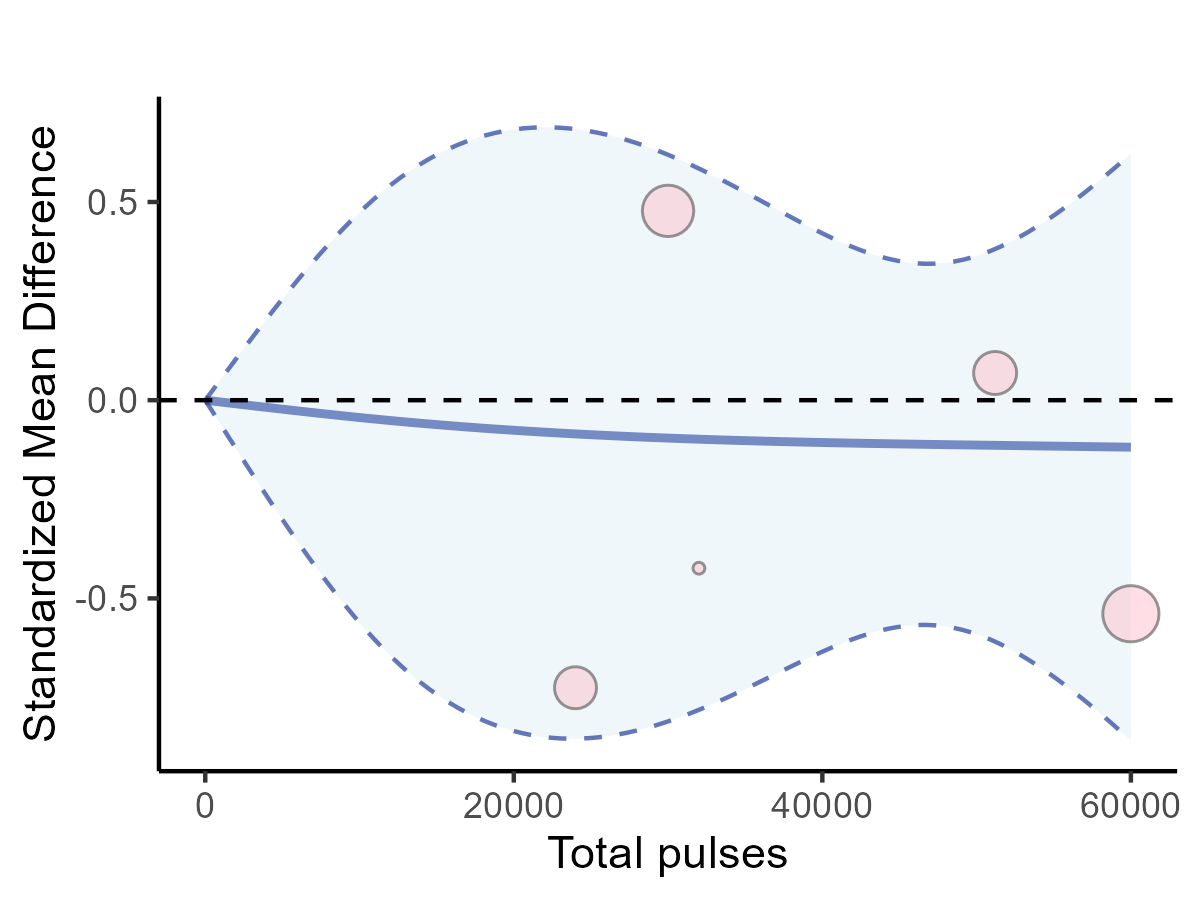
**

**44.eFigure 44. Dose-response relationship between Total pulses and improvement of ADAS-Cog.(Multiple Target Points)**

**
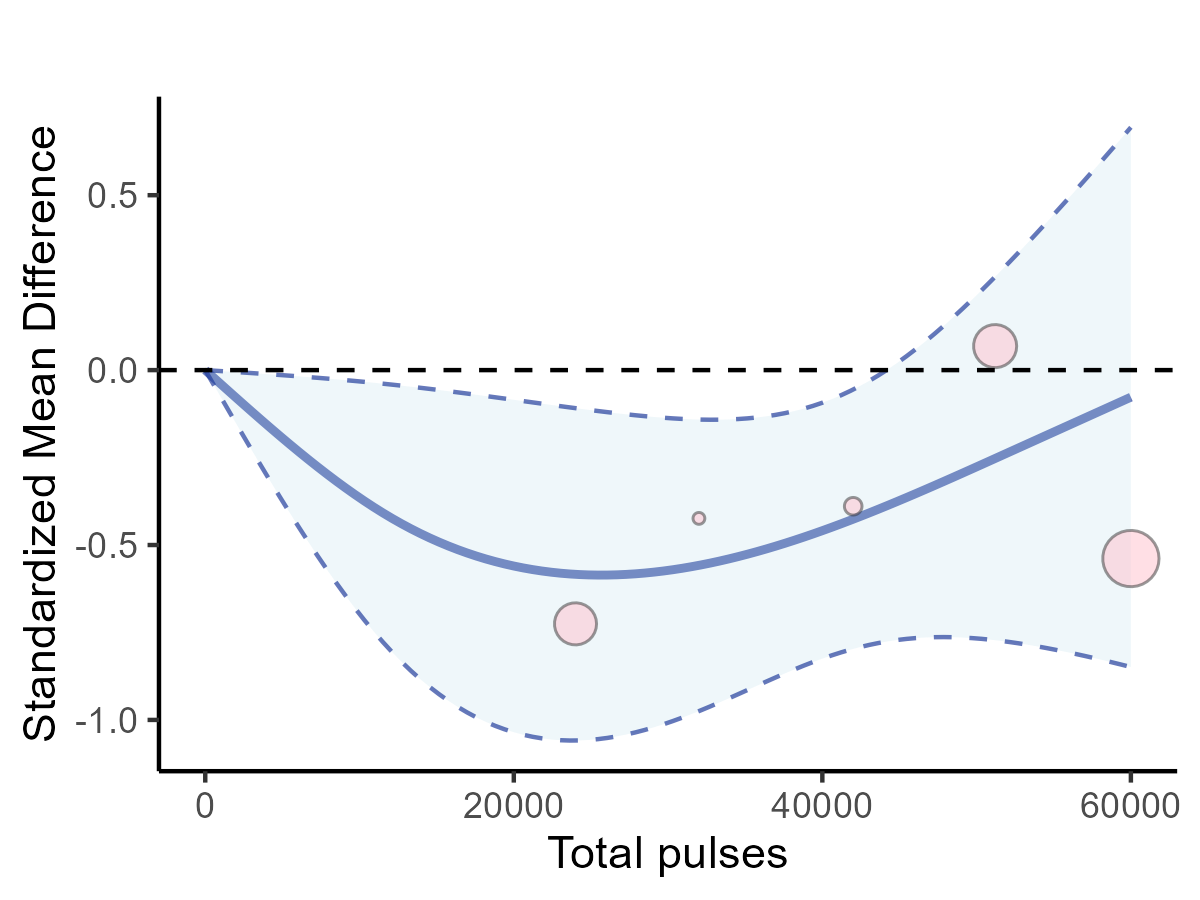
**

**Publication bias-funnel plot**

**45.Funnel plot of UPDRSⅢ**

**
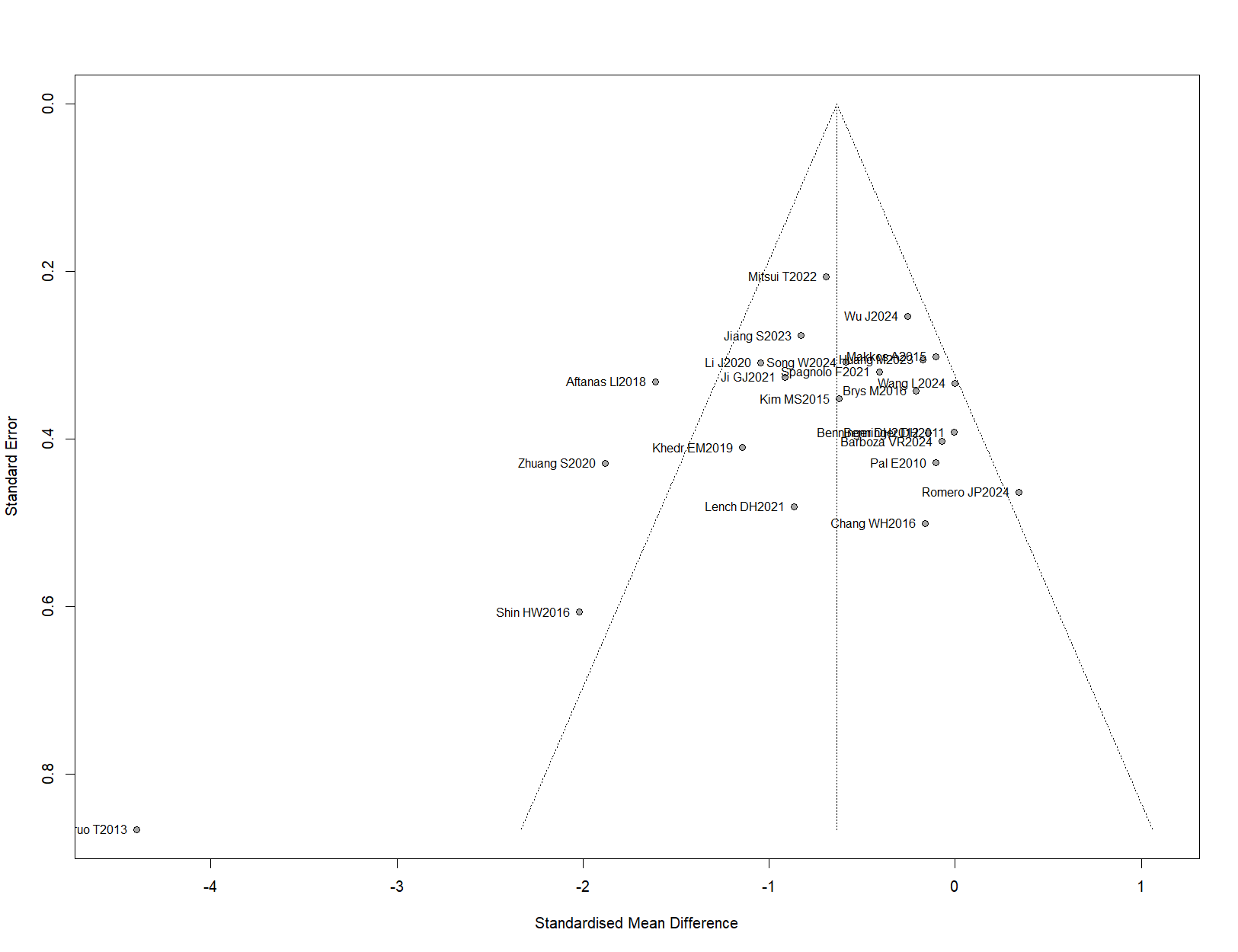
**

**46.Funnel plot of UPDRS**

**
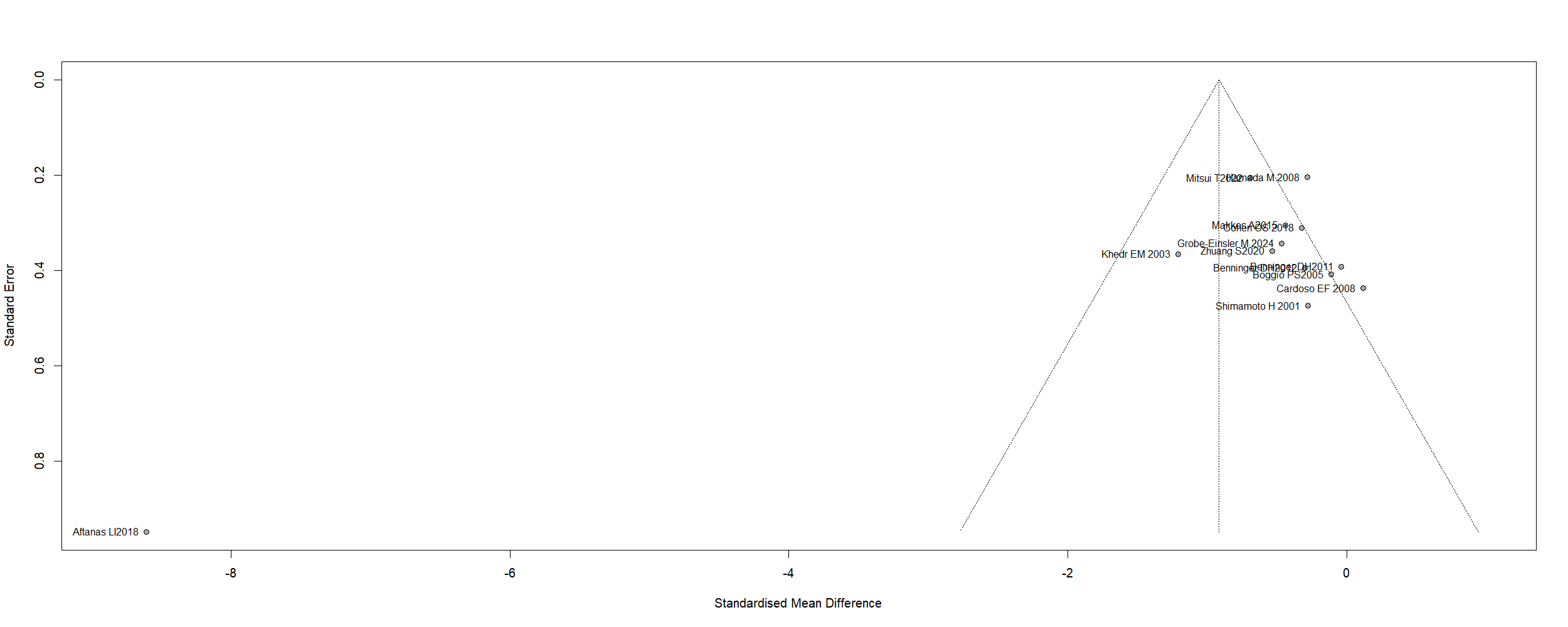
**

**47.Funnel plot of TUG**

**
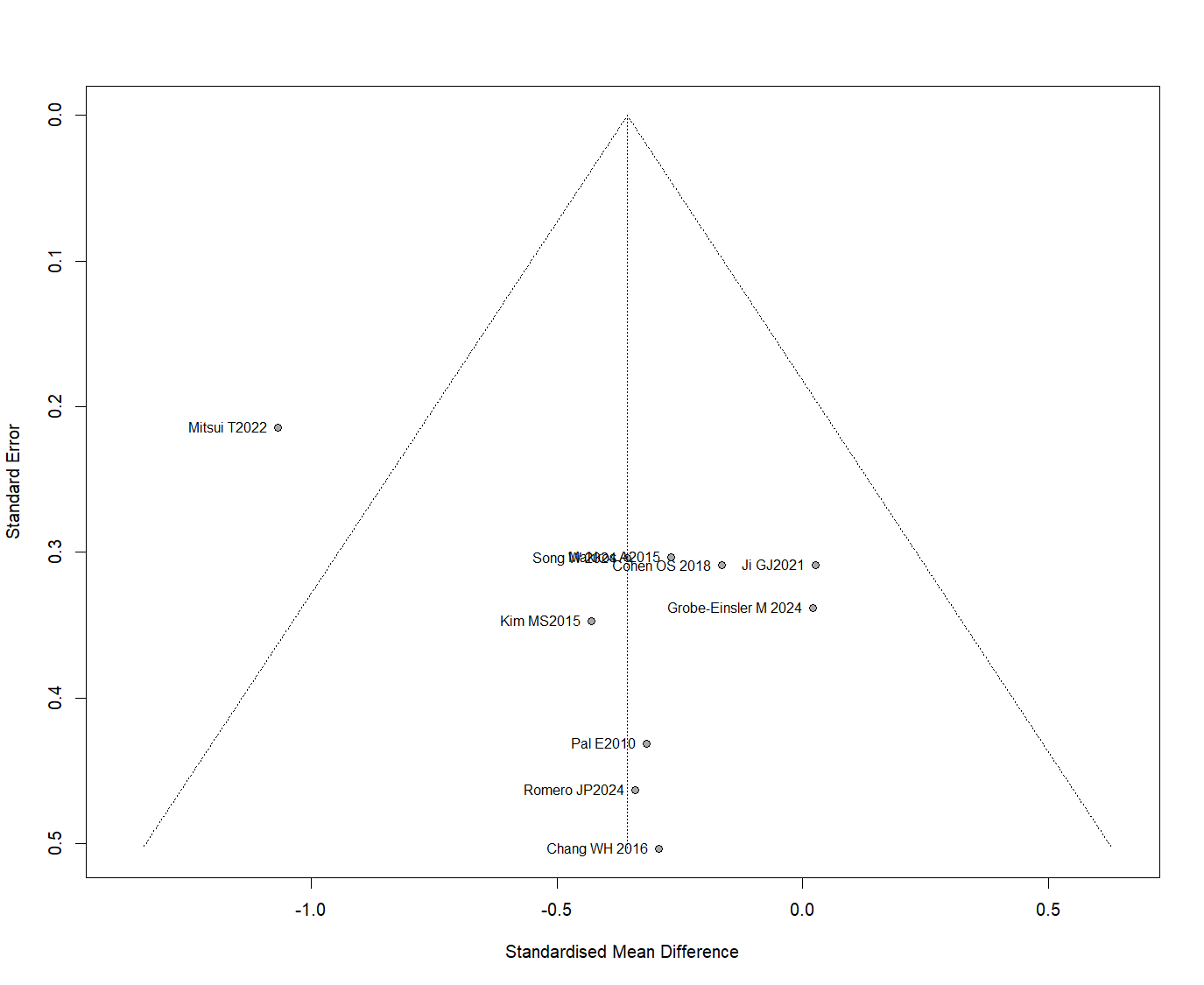
**

**48.Funnel plot of ADAS-Cog**

**
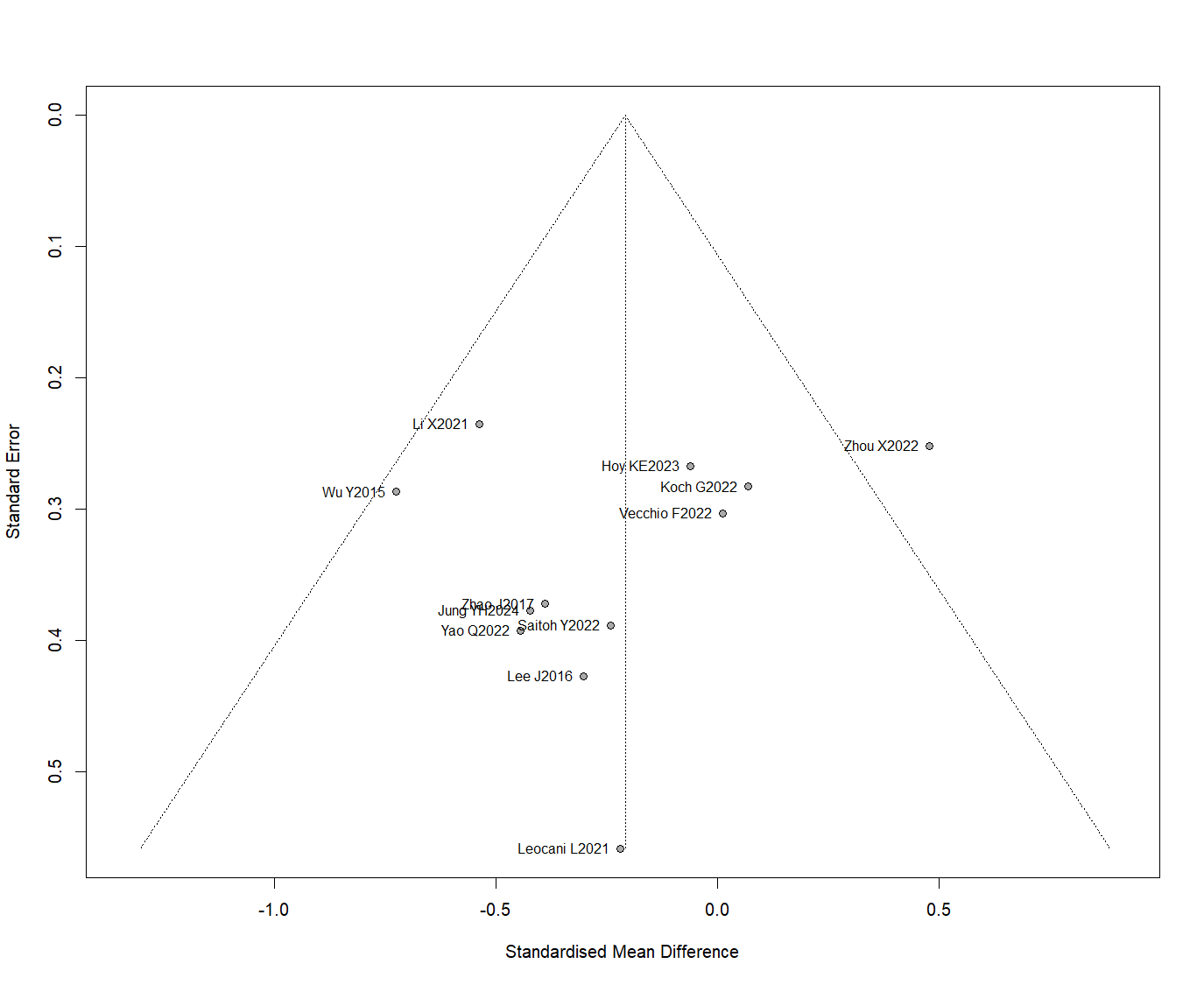
**

**48.Funnel plot of MMSE**

**
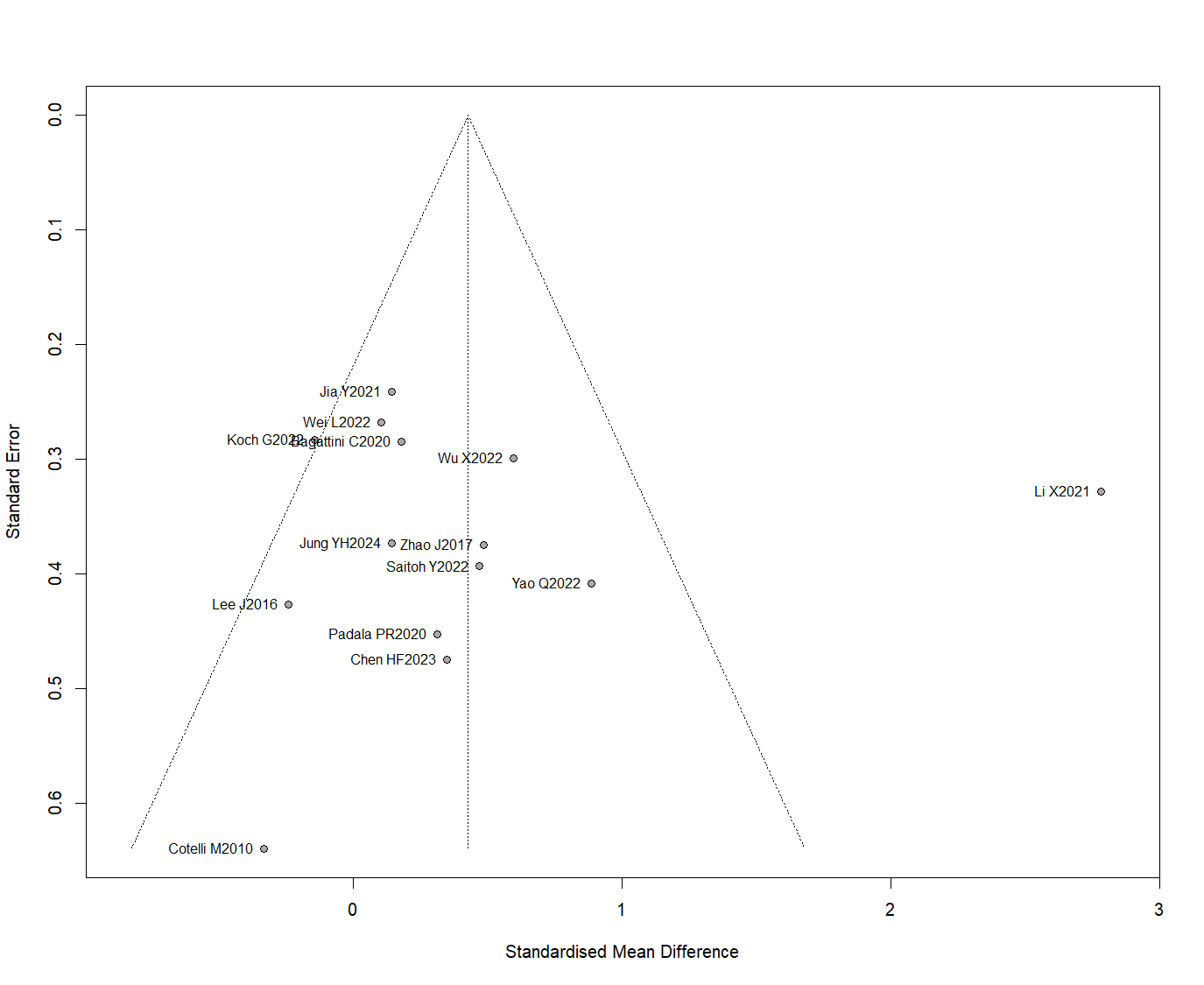
**
